# The Efficacy and Safety of Janus Kinase Inhibitors for Patients With COVID-19: A Living Systematic Review and Meta-Analysis

**DOI:** 10.3389/fmed.2021.800492

**Published:** 2022-01-27

**Authors:** Xueyang Zhang, Lianhan Shang, Guohui Fan, Xiaoying Gu, Jiuyang Xu, Yeming Wang, Lixue Huang, Bin Cao

**Affiliations:** ^1^School of Medicine, Tsinghua University, Beijing, China; ^2^Beijing University of Chinese Medicine, Beijing, China; ^3^Department of Pulmonary and Critical Care Medicine, Center of Respiratory Medicine, National Clinical Research Center for Respiratory Diseases, National Center for Respiratory Medicine, China-Japan Friendship Hospital, Beijing, China; ^4^Institute of Respiratory Medicine, Chinese Academy of Medical Sciences, Beijing, China; ^5^Institute of Clinical Medical Sciences, China-Japan Friendship Hospital, Beijing, China; ^6^Department of Pulmonary and Critical Care Medicine, Capital Medical University, Beijing, China; ^7^Tsinghua University-Peking University Joint Center for Life Sciences, Beijing, China

**Keywords:** COVID-19, SARS-CoV-2, Janus kinase inhibitors, baricitinib, systematic review, meta-analysis

## Abstract

**Background:**

Cytokine storm observed in patients with severe Coronavirus Disease 2019 (COVID-19) contributes to poor clinical outcomes and increased mortality. Janus kinases (JAKs) are important mediators in the cytokine storm. Therefore, we conduct a living systematic review and meta-analysis of the literature investigating efficacy and safety of JAK inhibitors for patients with COVID-19.

**Methods:**

Databases were searched up to December 1, 2021 for interventional and observational studies comparing JAK inhibitor treatment with concurrent control in patients with COVID-19. Efficacy and safety outcomes were evaluated by pooled risk ratio (RR).

**Results:**

Of 3,170 records retrieved, 15 studies were eligible and 13 were evaluated in the meta-analysis (*n* = 3,977). Based on data from three randomized controlled trials (RCTs), baricitinib treatment significantly decreased mortality by day 28 in hospitalized patients with COVID-19 (RR = 0.64, 95% CI 0.51–0.80) without increasing the incidence of adverse outcomes. In subgroup analysis, patients who required supplemental oxygen (RR = 0.62, 95% CI 0.41–0.95) or high-flow oxygen/non-invasive ventilation (RR = 0.59, 95% CI 0.42–0.85) at baseline benefited most. Pooled analysis of all eligible studies for JAK inhibitors (baricitinib, ruxolitinib, tofacitinib, and nezulcitinib) demonstrated a significant decrease in mortality (RR = 0.62, 95% CI 0.49–0.78) with no increase in the risk of adverse events.

**Conclusion:**

Baricitinib probably decreases mortality in hospitalized adult patients with COVID-19, especially for patients who required supplemental oxygen or high-flow oxygen/non-invasive ventilation at baseline. The efficacy and safety of other JAK inhibitors, such as ruxolitinib, tofacitinib, and nezulcitinib, await more evidence.

**Systematic Review Registration:**

https://www.crd.york.ac.uk/prospero/display_record.php?ID=CRD42021261414, identifier: CRD42021261414.

## Introduction

The past 2 years have witnessed the pandemic of Coronavirus Disease 2019 (COVID-19) induced by severe acute respiratory syndrome coronavirus 2 (SARS-CoV-2). During disease development, host immune response is illustrated to play a dual role (1). On the one hand, both innate and adaptive immune systems function to cope with the infection. Macrophages can be activated by SARS-CoV-2 to secrete type I and III interferons to promote antiviral responses in neighboring epithelial cells and interleukin-6 (IL-6) and IL-1β to recruit neutrophils and T lymphocytes (2, 3). Meanwhile, humoral immunity is stimulated to produce antibodies targeting at SARS-CoV-2, the high neutralizing potency of which was illustrated to be a predictor of survival (4). On the other hand, the dysregulated immune response could be pathological. Cytokine storm was well-described in patients with severe COVID-19, displaying significantly elevated serum levels of IL-6, IL-8, IL-10, IL-2R, and tumor necrosis factor-alpha (TNF-α) compared with those mild and moderate patients (5). Such a cytokine storm could result in tissue damage, vascular hemostasis disruption, anemia, and consequently lead to multi-organ failure (6).

Considering the critical role of hyperinflammation in the pathophysiology of COVID-19, much attention has been paid to anti-inflammation therapy (7), such as corticosteroids (8), IL-6 blocking agents (9), and Janus kinase (JAK) inhibitors (10).

Janus kinases are a family of important signaling mediators downstream of type I and II cytokine receptors (11). As tyrosine kinase, they can phosphorylate the tyrosine residues of signal transducers and activators of transcription (STATs) once activated, resulting in transcriptional regulation of target genes (12, 13). Based on this, researchers have been exploring the usage of JAK inhibitors in immune and inflammatory disease settings with the pathogenesis of type I and II cytokines. Up to now, several JAK inhibitors have been approved by U.S. FDA, such as baricitinib (14) and tofacitinib (15) for rheumatoid arthritis, and ruxolitinib (16) for myelofibrosis and polycythemia vera. As mentioned above, many cytokines, such as IL-6 and IL-10, were elevated in certain patients with COVID-19 (5), which could employ the JAK/STAT signaling pathway to exert their immunopathological effects (11). Therefore, it is worth to investigate the efficacy of JAK inhibitors in patients with COVID-19.

Clinical evidence investigating JAK inhibitor treatment for COVID-19 has been accumulating, but they reached an inconsistent conclusion in efficacy outcomes, especially for COVID-19 mortality and subgroup analysis on mortality according to baseline disease severity (17–19). Moreover, high-quality meta-analyses, which could better guide clinical practice of JAK inhibitors usage, such as optimal patient population, type of JAK inhibitors, and concomitant corticosteroids treatment, are absent. Thus, this systematic review aims to provide a comprehensive summary of current evidence for a better understanding of the efficacy and safety of JAK inhibitors for COVID-19.

## Materials and Methods

### Search Strategy and Selection Criteria

This study was conducted following the Preferred Reporting Items for Systematic Reviews and Meta-Analyses (PRISMA; Appendix 1) statement, and the protocol of this systematic review was registered in PROSPERO (CRD42021261414; Appendix 2).

Inclusion criteria of studies were randomized controlled trials (RCT), non-randomized clinical trials, or observational studies with a concurrent control that investigated the efficacy and/or safety of JAK inhibitor treatment in patients with COVID-19, with no limitations on publication status or language. Exclusion criteria were (I) study designs other than the ones mentioned above; (II) meeting abstracts and articles with no full texts available; and (III) studies not providing sufficient information to be included in the systematic review. One standard existed additionally for studies to be included in the meta-analysis: the control group should be either placebo or standard-of-care group.

The databases we searched included MEDLINE (*via* Ovid), Embase, The Cochrane Central Register of Controlled Trials (CENTRAL), China National Knowledge Infrastructure (CNKI), Wanfang database, SinoMed, World Health Organization (WHO) COVID-19 database (global literature on coronavirus disease; https://search.bvsalud.org/global-literature-on-novel-coronavirus-2019-ncov/), and the Cochrane COVID-19 study register. We also hand-searched three pre-print servers (MedRxiv, BioRxiv, and SSRN). The search strategy was built based on terms related to COVID-19, SARS-CoV-2, and JAK inhibitors, and details are present in Appendix 3. Search for this version of the systematic review was first conducted on June 11, 2021 and updated until December 1, 2021. The final analysis of this manuscript was based on articles retrieved from the search on December 1, 2021.

Two investigators (X Z and L S) independently performed literature screening *via* Endnote 20 for eligible studies. Any disagreement was resolved through discussion.

### Data Extraction and Risk of Bias Assessment

Two investigators (X Z and L S) independently performed data extraction and risk of bias assessment. Any disagreement was resolved through discussion. Data extracted mainly included authors, publication time, study design, study location, inclusion and exclusion criteria, sample size, intervention (type of JAK inhibitors, dose, route of administration, frequency, duration, and other concurrent treatment), treatment of control group, and key efficacy and safety outcomes.

Efficacy outcomes evaluated in this version were (I) COVID-19 mortality, (II) the incidence of invasive mechanical ventilation (IMV), and (III) time to recovery. As for safety, outcomes that include (I) adverse events, (II) serious adverse events, and (III) infection or secondary infection were analyzed.

The risk of bias assessment for RCTs was conducted adopting Cochrane RoB 2.0 tool (RoB 2) (20) and visualized using the robvis tool (21–23). As for observational studies, the Newcastle-Ottawa scale (NOS) was employed, and studies equal to or more than the score of five were qualified for meta-analysis (24).

### Data Analysis

In the meta-analysis, pooled risk ratio (RR) with 95% confidence interval (CI) was adopted for dichotomous data, which was generated based on raw events number and total number extracted from included studies using the Mantel-Haenszel method. For time to recovery, calculating pooled mean difference was not feasible due to the studies provided median with 95% CI, thus pooled RR was generated from RR and 95% CI extracted from the original studies. Considering potential heterogeneity across studies, the random-effects model was used for all outcomes. Statistical heterogeneity was assessed using the *I*^2^ and Q statistic, publication bias was described by funnel plot, and sensitivity analysis was performed by omitting individual studies. For COVID-19 mortality, subgroup analysis was conducted according to baseline score on the National Institute of Allergy and Infectious Diseases (NIAID) ordinal scale. All analyses mentioned above were performed using the R package “meta” (version 4.18-1; R Foundation for Statistical Computing) (25).

### Living Systematic Review

As a living systematic review and meta-analysis, searches on databases will be conducted monthly. If important evidence, which may potentially change previous conclusions, is published, this study will be updated in time.

## Results

### Study Selection and Characteristics

**Figure 1:**
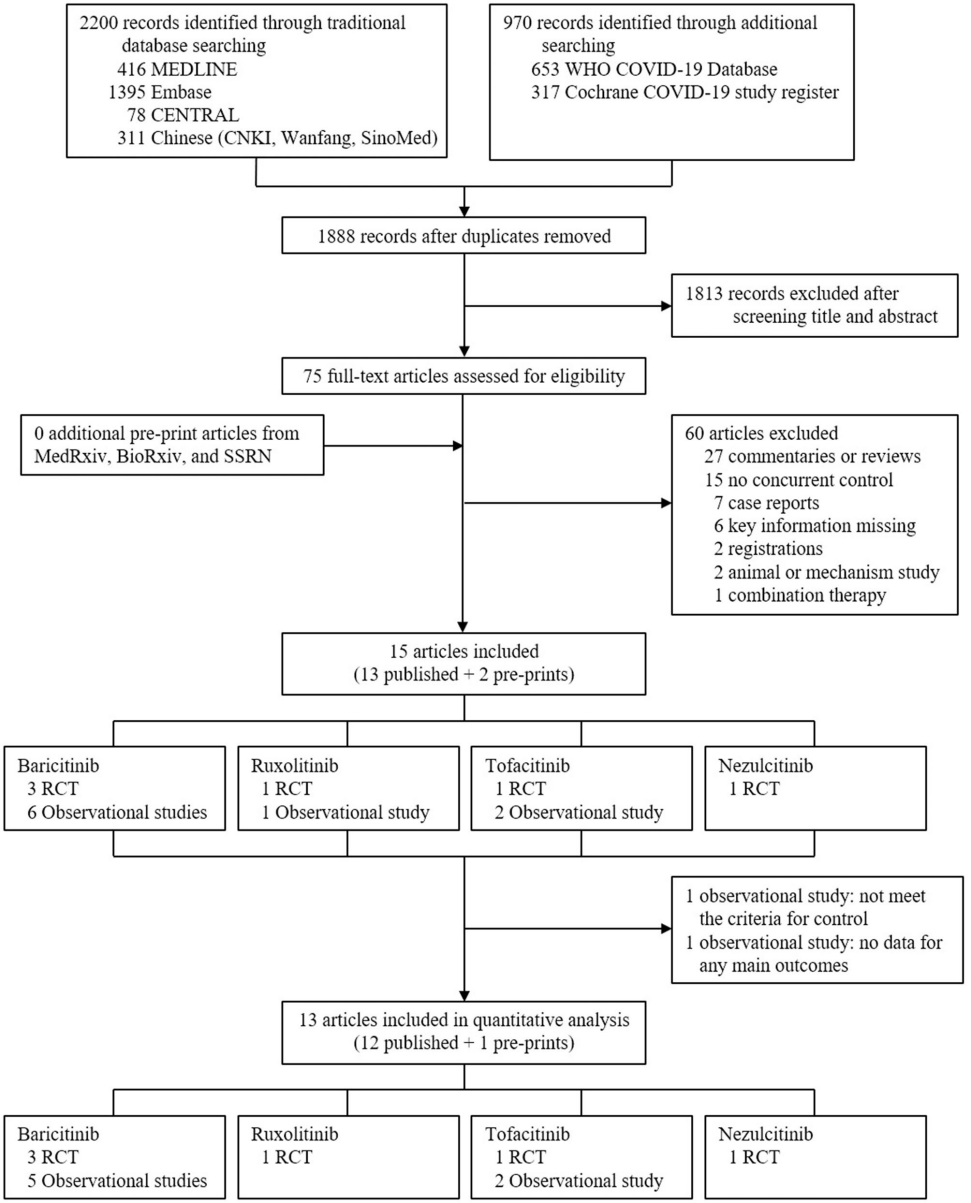
Study selection. CENTRAL, Cochrane Central Register of Controlled Trials; CNKI, China National Knowledge Infrastructure; WHO, World Health Organization; COVID-19, coronavirus disease 2019; RCT, randomized controlled trial.

In total, 3,170 records were retrieved initially after searching databases, with 75 articles remaining after removing duplicates and screening titles and abstracts to exclude irrelevant studies. After reading the full texts of the 75 articles, 15 were included in the systematic review (Figure 1) (17–19, 26–37). In the risk of bias assessment, no RCTs were at high risk of bias, and all observational studies scored at least six according to ROB 2 and NOS, respectively (Supplementary Figure S1 and Supplementary Table S1). Based on information extracted, 13 studies were evaluated in the meta-analysis (Figure 1), i.e., six RCTs [three for baricitinib (17–19), one for ruxolitinib (32), one for tofacitinib (34), and one for nezulcitinib (37)], and seven observational studies [five for baricitinib (26, 27, 29–31) and two for tofacitinib (35, 36)]. Results from the remaining two studies [one observational study for baricitinib (28), and one observational study for ruxolitinib (33)] would be narratively described.

The main characteristics of 15 included studies are summarized in Table 1. Particularly, two RCTs (17, 18) for baricitinib had relatively large sample sizes (1,033 and 1,525, respectively), while the remaining ones were all <350. Participants were all hospitalized patients with COVID-19, but the specific inclusion criteria varied in many aspects, such as severity and inflammatory marker level. For efficacy outcomes analyzed in this study (i.e., mortality, the incidence of IMV, and time to recovery), 13, 7, and 2 studies provided related data, respectively. Regarding safety, six RCTs (17–19, 32, 34, 37) displayed detailed information on adverse events and serious adverse events, while five RCTs (17–19, 32, 34) plus two observational studies (29, 31) reported the incidence of infection or secondary infection.

**Table 1:** Characteristics of studies included in the systematic review.

**Study**	**JAK inhibitor**	**Study design and setting**	**Main inclusion criteria and enrollment period**	**Intervention**	**Control**	**Key outcomes**	**Main findings**
Kalil et al. (17)	Baricitinib	RCT 67 centers in 8 countries	Hospitalized adult (≥18 years) patients with moderate or severe COVID-19. 2020.05.28-2020.07.01	Baricitinib 4 mg, PO, QD, 14 days or until hospital discharge (2 mg for eGFR <60ml/min/1.73m^2^). Remdesivir. Standard of care. *n* = 515	Placebo. Remdesivir. Standard of care. *n* = 518	Time to recovery. Clinical status at day 15. Mortality by day 28. Duration of hospitalization. Incidence and duration of each type of respiratory support.	The addition of baricitinib to remdesivir reduced recovery time and accelerated clinical status improvement, but did not reduce mortality in moderate or severe patients.
Marconi et al. (18)	Baricitinib	RCT 101 centers in 12 countries	Hospitalized adult (≥18 years) patients with COVID-19 with NIAID ordinal score of 4–6. At least one elevated inflammatory marker (CRP, D-Dimer, LDH, and ferritin). 2020.06.11–2021.01.15	Baricitinib 4 mg, PO, QD, 14 days or until hospital discharge (2 mg for eGFR ≥ 30 to <60 ml/min/1.73m^2^). Standard of care. *n* = 764	Placebo. Standard of care. *n* = 761	Proportion of patients having progressed to NIAID ordinal score of 6–8 by day 28. Mortality by day 28. Time to recovery. Duration of hospitalization.	Baricitinib showed no significant reduction in the frequency of disease progression overall, but reduced mortality in patients with NIAID ordinal score of 4–6.
Ely et al. (19)	Baricitinib	RCT 18 centers in 4 countries	Hospitalized adult (≥18 years) patients with COVID-19 with NIAID ordinal score of 7. At least one elevated inflammatory marker (CRP, D-Dimer, LDH, and ferritin). 2020.12.23–2021.04.10	Baricitinib 4 mg, PO, QD, 14 days or until hospital discharge (2 mg for eGFR ≥ 30 to <60 ml/min/1.73m^2^). Standard of care. *n* = 51	Placebo. Standard of care. *n* = 50.	Mortality by day 28 and 60. Clinical status. Time to recovery. Duration of hospitalization.	Baricitinib plus standard of care predominantly including corticosteroids reduced mortality by day 28 and 60 in patients with NIAID ordinal score of 7.
Bronte et al. (26)	Baricitinib	Observational study 2 centers in Italy	Hospitalized adult (≥18 years) patients with COVID-19 with symptoms onset not exceeding 9 days. Interstitial lung involvement not exceed 50% on chest x-ray or CT. 2020.03.25–2020.04.18	Baricitinib 4 mg, PO, Bid, 2 days; then QD, 7 days (2 mg for patients older than 75 years or with GFR <30 mL/min/1.73 m^2^, hepatotoxicity, or myelotoxicity). Either hydroxychloroquine or antiviral therapy (lopinavir/ritonavir) or in combination. Standard of care. *n* = 20	Hydroxychloroquine or antiviral therapy (lopinavir/ritonavir) or in combination. Standard of care. *n* = 56	Mortality. Incidence of ARDS. Duration of hospitalization. Level of systemic inflammation.	Baricitinib reduced level of systemic inflammation and mortality in hospitalized patients.
Rosas et al. (27)	Baricitinib	Observational study 1 center in Spain	Hospitalized adult (≥18 years) patients with COVID-19 with PaO_2_/FiO_2_ <300 mmHg. Interstitial pneumonia. 2020.03.27–2020.04.02	Baricitinib 2 mg or 4 mg, PO, QD. With (*n* = 12) or without (*n* = 11) tocilizumab. Standard of care. *n* = 23	With (*n* = 20) or without (*n* = 17) tocilizumab. Standard of care. *n* = 37	Mortality by day 30. Incidence of ICU admission.	Baricitinib did not cause serious side effects in COVID-19 patients with interstitial pneumonia.
Stebbing et al. (28)	Baricitinib	Observational study 1 center in Italy and 1 center in Spain	Hospitalized patients with moderate-to-severe or severe COVID-19 with SaO_2_ <94% and not on mechanical ventilation. Italy: PaO2/FiO2 ratio <300 mmHg. 2020.03.07–2020.03.31 Spain: ≥70 years. 2020.03.09–2020.04.20	Baricitinib Italy: 4 mg, PO, QD, 14 days. Spain: 2 mg or 4 mg, PO, QD, 3–11 days. Standard of care. *n* = 83	Standard of care. *n* = 83	Incidence of death or IMV (composite outcome).	Baricitinib reduced the incidence of death or IMV (composite outcome) in moderate-to-severe or severe patients.
Pérez-Alba et al. (29)	Baricitinib	Observational study 1 center in Mexico	Hospitalized adult (>18 years) patients with severe COVID-19 requiring supplemental oxygen. 2020.03–2020.11	Baricitinib 4 mg, PO, QD, 14 days or until hospital discharge (2 mg for eGFR ≥ 30 to <60 ml/min/1.73 m^2^). Dexamethasone. Standard of care. *n* = 123	Dexamethasone. Standard of care. *n* = 74	Mortality by day 30. Incidence of IMV. Incidence of ICU admission. Duration of hospitalization.	The addition of baricitinib to dexamethasone reduced mortality but not the incidence of IMV in patients with severe COVID-19.
Abizanda et al. (30)	Baricitinib	Observational study 1 center in Spain	Hospitalized patients with moderate-to-severe or severe COVID-19 not requiring mechanical ventilation. 2020.03.09–2020.07.07	Baricitinib (regimen NA). Standard of care. *n* = 164	Standard of care. *n* = 164	Mortality by day 30.	Baricitinib reduced mortality in patients with moderate-to-severe or severe COVID-19.
Masiá et al. (31)	Baricitinib	Observational study 1 center in Spain	Hospitalized patients with COVID-19 having received corticosteroids and tocilizumab and requiring high-flow nasal cannula or non-invasive mechanical ventilation. 2020.03.01–2021.03.31	Baricitinib (regimen NA). Standard of care. *n* = 95	Standard of care. *n* = 95	Mortality by day 28, 60, and 90. Incidence of death or IMV (composite outcome). Viral load. Change of biomarkers.	The addition of baricitinib to corticosteroids and tocilizumab did not reduce mortality in hospitalized patients.
Cao et al. (32)	Ruxolitinib	RCT 3 centers in China	Hospitalized adult (18–75 years) patients with severe COVID-19 and not on IMV. 2020.02.09–2020.02.28	Ruxolitinib 5 mg, PO, Bid. Standard of care. *n* = 20	Placebo (100 mg vitamin C). Standard of care. *n* = 21	Time to clinical improvement. Clinical improvement rate. Mortality by day 28. Duration of hospitalization. Virus clearance time. Time to lymphocyte recovery.	Ruxolitinib trended toward improving clinical status faster in severe patients.
Stanevich et al. (33)	Ruxolitinib	Observational study 4 centers in Russia	Hospitalized adult patients with COVID-19 with NIAID ordinal score of 5–6. Enrollment period: NA.	Ruxolitinib 5–10 mg, PO, Bid, until oxygen support withdrawal. Standard of care. *n* = 146	Dexamethasone. Standard of care. *n* = 146	Mortality.	Ruxolitinib was comparable to dexamethasone in mortality of patients with NIAID ordinal score of 5–6.
Guimarães et al. (34)	Tofacitinib	RCT 15 centers in Brazil	Hospitalized adult (≥18 years) patients with COVID-19 with hospitalization for <72 h and not on non-invasive ventilation, IMV or ECMO. 2020.09.16–2020.12.13	Tofacitinib 10 mg, PO, Bid, 14 days or until hospital discharge (5 mg for eGFR <50 ml/min/1.73 m^2^ or with some other conditions). Standard of care. *n* = 144	Placebo. Standard of care. *n* = 145	Incidence of death or respiratory failure (composite outcome). Mortality by day 28. Clinical status.	Tofacitinib reduced the incidence of death or respiratory failure (composite outcome) in hospitalized patients.
Maslennikov et al. (35)	Tofacitinib	Observational study 1 center in Russia	Hospitalized adult (>18 years) patients with COVID-19. CRP 60–150 mg/L. 2020.04–2020.07	Tofacitinib 10 mg, PO, Bid, 1 day; then 5 mg, PO, Bid, 4 days. NO other anti-cytokine therapy. Standard of care. *n* = 32	NO anti-cytokine therapy. Standard of care. *n* = 30	Mortality by day 50. Duration of hospitalization. Duration of disease. Incidence of ICU admission and mechanical ventilation. Change of key biomarkers, chest CT, and respiratory function.	Tofacitinib reduced level of systemic inflammation in hospitalized patients.
Singh et al. (36)	Tofacitinib	Observational study 1 center in India	Hospitalized patients with severe COVID-19 and NIAID ordinal score of 4–6. 2021.04.08–2021.05.10	Tofacitinib 10 mg, PO, Bid. Dexamethasone and anticoagulants. Standard of care. *n* = 25	Dexamethasone and anticoagulants. Standard of care. *n* = 25	Clinical status. Mortality by day 21. Incidence of IMV. Oxygenation.	Tofacitinib reduced intubation rates and prevented clinical worsening, but did not reduce mortality in patients with NIAID ordinal score of 4–6.
Singh et al. (37)	Nezulcitinib	RCT Centers in Moldova, UK and Ukraine	Hospitalized adult (18–80 years) patients with COVID-19 (symptoms for 3–14 days) requiring supplemental oxygen. NOT receiving other JAK inhibitors or anti-IL-6 therapy. Enrollment period: NA.	Nezulcitinib 2 mg, inhaled, QD, 1 day; then 1 mg for up to 6 days. (*n* = 6) Or 6mg, inhaled, QD, 1 day; then 3mg for up to 6 days. (*n* = 7) Or 10mg, inhaled, QD, up to 7 days. (*n* = 6) Standard of care. *n* = 19	Inhaled placebo. Standard of care. *n* = 6	Mortality by day 28. Clinical status. Duration of hospitalization. Oxygenation.	Nezulcitinib trended toward improving clinical status and decreasing mortality in patients requiring supplemental oxygen.

*ARDS, acute respiratory distress syndrome; Bid, twice daily; COVID-19, Coronavirus Disease 2019; CRP, C-reactive protein; CT, computed tomography; ECMO, extracorporeal membrane oxygenation; eGFR, estimated glomerular filtration rate; FiO_2_, fraction of inspired oxygen; ICU, intensive care unit; IL-6, interleukin 6; IMV, invasive mechanical ventilation; JAK, Janus kinase; LDH, Lactate dehydrogenase; NA, not available; NIAID, the National Institute of Allergy and Infectious Diseases; NIAID ordinal score of 4, hospitalized, not requiring supplemental oxygen, requiring ongoing medical care; NIAID ordinal score of 5, hospitalized, requiring supplemental oxygen; NIAID ordinal score of 6, hospitalized, on high-flow oxygen devices or non-invasive ventilation; NIAID ordinal score of 7, hospitalized, on mechanical ventilation or ECMO; NIAID ordinal score of 8, death; PaO_2_, partial pressure of oxygen; PO, by mouth; QD, once daily; RCT, randomized controlled trial; SaO_2_, blood oxygen saturation*.

### Efficacy and Safety of Baricitinib in Patients With COVID-19

**Figure 2:**
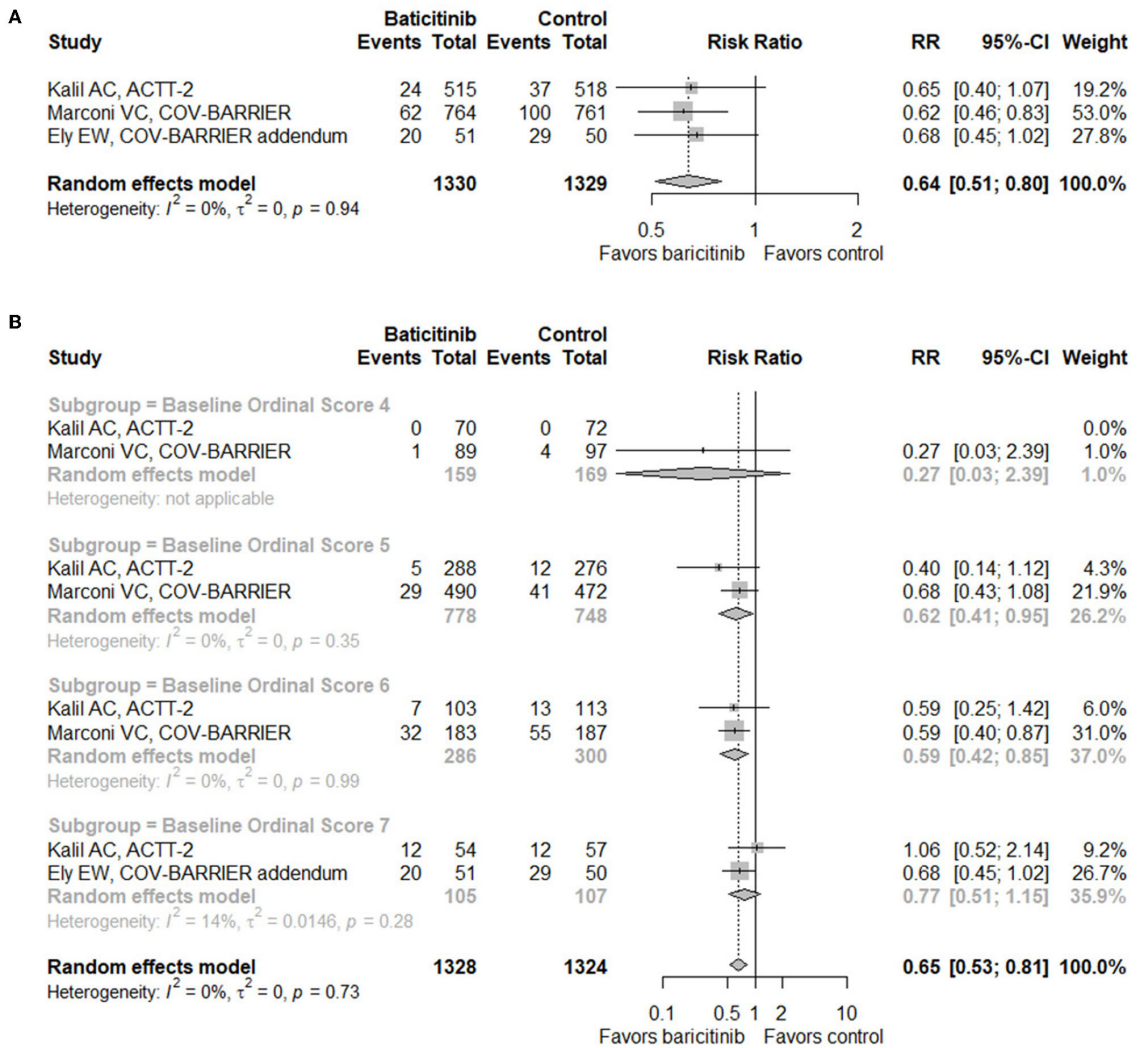
Forest plots for mortality. **(A)** Forest plot for mortality with baricitinib vs. control in randomized controlled trials. **(B)** Subgroup analysis for mortality with baricitinib vs. control in randomized controlled trials according to the baseline NIAID ordinal scale score. Baseline scores on the NIAID ordinal scale of 7 subjects were missing in the COV-BARRIER trial. RR, risk ratio; CI, confidence interval; NIAID, the National Institute of Allergy and Infectious Diseases.

Based on data from three RCTs (*n* = 2,659) (17–19), baricitinib treatment demonstrated a significant decrease of mortality by day 28 in hospitalized patients with COVID-19 (RR = 0.64, 95% CI 0.51–0.80; Figure 2A). When conducting subgroup analysis according to baseline score on the eight-level NIAID ordinal scale which reflects disease severity (Figure 2B), patients with a score of six (hospitalized, on high-flow oxygen devices, or non-invasive ventilation; RR = 0.59, 95% CI 0.42–0.85) benefited most from baricitinib in terms of mortality by day 28, followed by patients with a score of five (hospitalized, requiring supplemental oxygen; RR = 0.62, 95% CI 0.41–0.95). On the contrary, benefits were not observed in patients with a score of four (hospitalized, not requiring supplemental oxygen, requiring ongoing medical care; RR = 0.27, 95% CI 0.03–2.39) or seven (hospitalized, requiring IMV, or extracorporeal membrane oxygenation [ECMO]; RR = 0.77, 95% CI 0.51–1.15). In addition, when taking both RCTs and observational studies into consideration, the mortality of the baricitinib group was still significantly lowered compared with the control group (RR = 0.66, 95% CI 0.50–0.86; Supplementary Figure S2A).

As for the incidence of IMV, one RCT (17) and two observational studies (29, 31) reported related data, showing no significant difference between the two groups (RR = 1.03, 95% CI 0.51–2.10; Supplementary Figure S2B). For time to recovery, defined as a score on the NIAID ordinal scale of 1–3, the baricitinib treatment group exhibited a faster recovery (RR = 1.13, 95% CI 1.04–1.23; Supplementary Figure S2C). When we looked at the raw data, however, we found that the median time to recovery was 7 vs. 8 d in the ACTT-2 trial (17) and 10 vs. 11 d in the COV-BARRIER trial (18), indicating that absolute change of time to recovery was relatively small.

**Figure 3:**
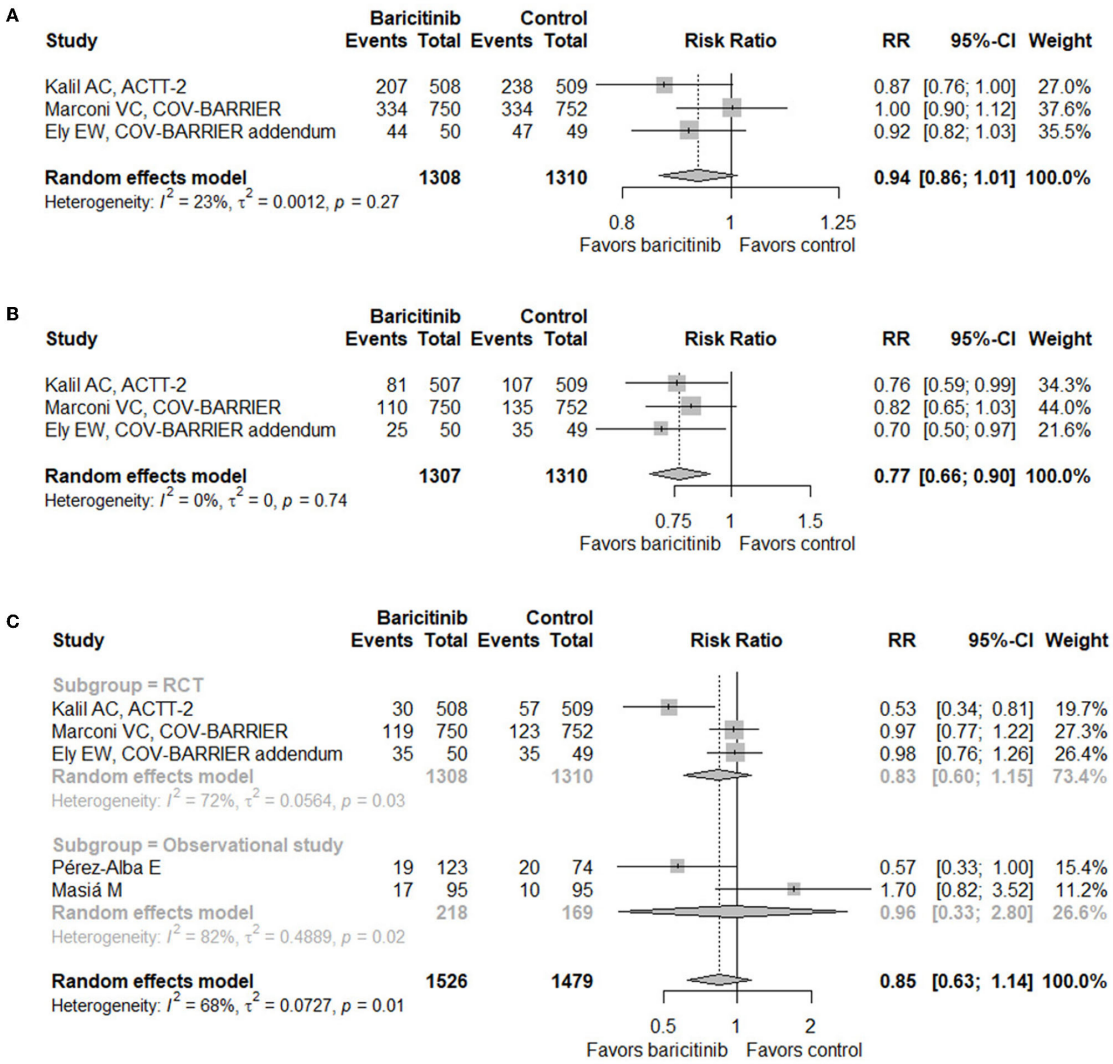
Forest plots for safety outcomes with baricitinib vs. control. **(A)** Adverse events. **(B)** Serious adverse events. **(C)** Infection or secondary infection. RR, risk ratio; CI, confidence interval; RCT, randomized controlled trial.

When it comes to safety, three RCTs (17–19) and two observational studies (29, 31) provided relevant data. Baricitinib treatment did not increase the incidence of adverse events (RR = 0.94, 95% CI 0.86–1.01; Figure 3A), serious adverse events (RR = 0.77, 95% CI 0.66–0.90; Figure 3B), or infection or secondary infection (RR = 0.85, 95% CI 0.63–1.14; Figure 3C).

### Efficacy and Safety of Ruxolitinib, Tofacitinib, and Nezulcitinib in Patients With COVID-19

One RCT (34) and two observational studies (35, 36) were included for tofacitinib, showing a significant decrease in short-term mortality (RR = 0.46, 95% CI 0.25–0.88; Supplementary Figure S3A) and the incidence of IMV (RR = 0.36, 95% CI 0.17–0.80; Supplementary Figure S3C). Particularly, in the RCT study for tofacitinib (34), subgroup analysis for mortality was also conducted according to baseline score on the NIAID ordinal scale (Supplementary Figure S3B). Limited by sample size, no benefit was observed in any subgroup. Besides, it should be noticed that patients requiring non-invasive ventilation (part of patients scoring six on the NIAID ordinal scale) were excluded from the study, which is to say that the subgroup with a score of six was not representative enough. As for safety outcomes, only the RCT provided related data, exhibiting no change of the incidences (Supplementary Figures S3D–F).

When it comes to ruxolitinib and nezulcitinib, only one RCT was included with a small sample size (*n* = 41 and 25, respectively) for each of them [(32, 37); Supplementary Figures S4, S5]. Thus, convincing conclusions could not be drawn from the present evidence.

One observational study of ruxolitinib (33) not evaluated in the meta-analysis compared mortality between the ruxolitinib treatment group and the dexamethasone treatment group, demonstrating that these two groups were comparable in terms of mortality (9.6 vs. 13.0%, superiority *p* = 0.35, non-inferiority *p* = 0.0137). This study indicated that ruxolitinib might be an alternative therapy for patients with contradictions of corticosteroids.

### Efficacy and Safety of JAK Inhibitors in Patients With COVID-19

**Figure 4:**
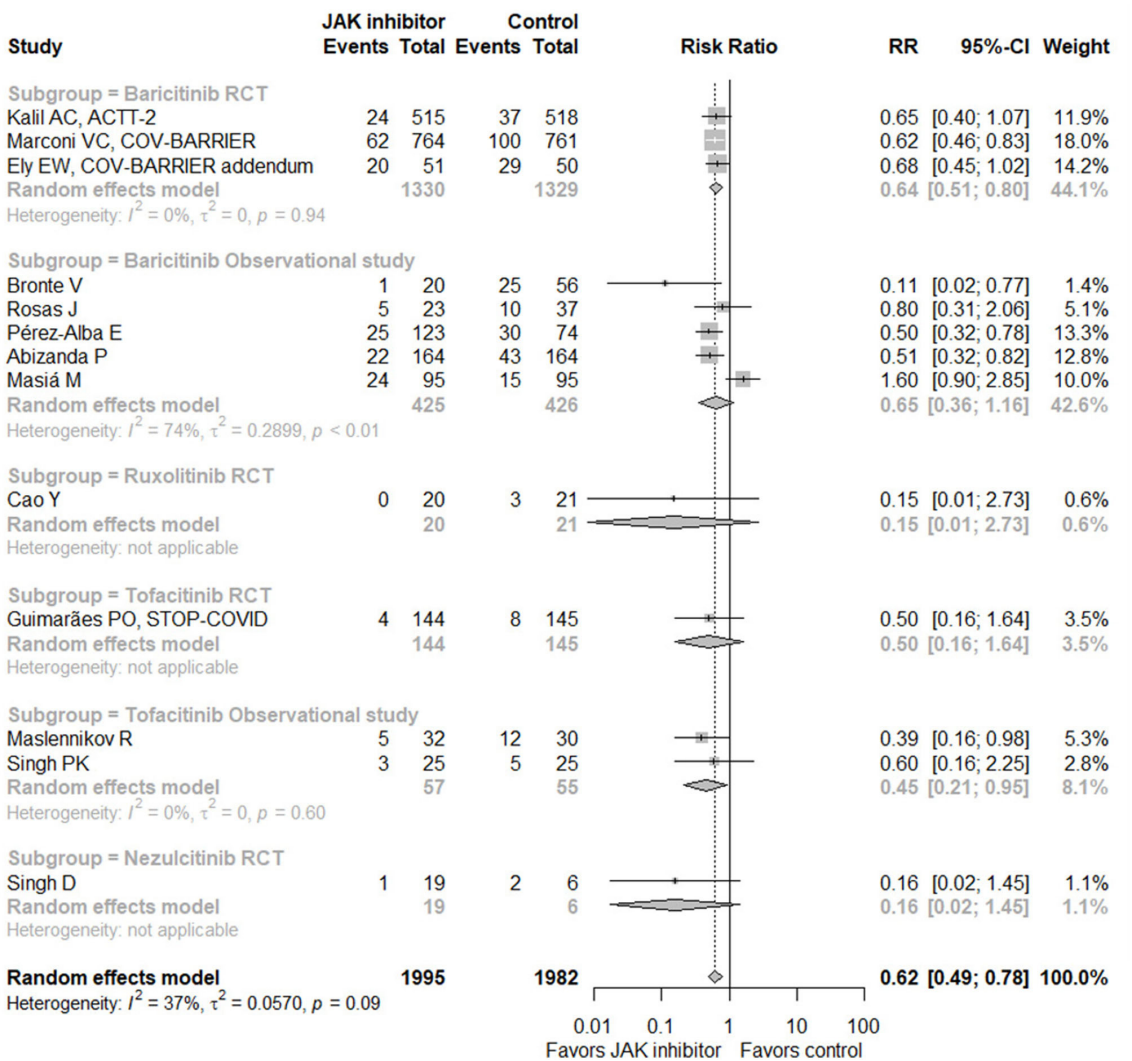
Forest plot for mortality with Janus kinase inhibitor vs. control in all eligible studies. JAK, Janus kinase; RR, risk ratio; CI, confidence interval; RCT, randomized controlled trial.

When taking all eligible studies (13 studies with 3,977 subjects) (17–19, 26, 27, 29–32, 34–37) for meta-analysis into consideration, JAK inhibitor treatment could significantly decrease mortality in hospitalized patients with COVID-19 compared with control (RR = 0.62, 95% CI 0.49–0.78; Figure 4). Of the 13 studies, seven provided data of the incidence of IMV, with pooled RR of 0.63 (95% CI 0.34–1.17), suggesting no significant difference (Supplementary Figure S6A). In terms of safety, six RCTs (17–19, 32, 34, 37) and two observational studies (29, 31) displayed related data, showing no increase of adverse events, serious adverse events, or infection or secondary infection caused by JAK inhibitor treatment (Supplementary Figures S6B–D). Sensitivity analysis by omitting any single study exhibited similar results (Supplementary Figure S7). Due to the small number of eligible studies, funnel plots could only give some indications for potential bias of publication (Supplementary Figure S8).

## Discussion

In the first version of this living systematic review and meta-analysis that includes six RCTs and nine observational studies up to December 1, 2021, baricitinib was shown to reduce short-term mortality of hospitalized patients with COVID-19 with no increase in adverse outcomes based on analysis of three RCTs, and patients would benefit more if their baseline score on the NIAID ordinal scale was equal to five (hospitalized, requiring supplemental oxygen) or six (hospitalized, on high-flow oxygen devices, or non-invasive ventilation). Analysis of one RCT and two observational studies demonstrated the potential efficacy of tofacitinib in short-term mortality and the incidence of IMV. Due to the limited number of studies and sample size, conclusions could not be drawn on the efficacy of ruxolitinib and nezulcitinib.

While direct viral damage may dominate at the early stage of COVID-19, immune dysfunction and hyperinflammatory damage are more important at later/severe stages (38). Theoretically, JAK inhibitors function in patients with COVID-19 by blocking the inflammatory effect of type I and II cytokines elevated systematically (11). One study (39) collected whole blood from patients with COVID-19 and treated it with SARS-CoV-2-derived peptide *in vitro*. When adding baricitinib, the inflammatory response stimulated by SARS-CoV-2 was curtailed, illustrated as significantly downregulated levels of IL-1β, IL-6, and TNF-α, etc. Additionally, in a SARS-CoV-2-infected rhesus macaque model (40), baricitinib was proved to decrease neutrophils and macrophages infiltration and activation in the lung, leading to limited pulmonary lesions. The evidence above supported the efficacy of JAK inhibitors in patients with COVID-19 through suppressing hyperinflammation. Thus, patients with increased inflammatory markers, such as C-reactive protein (CRP), possibly benefit more from JAK inhibitor treatment. Elevated inflammatory marker was one of the inclusion criteria of several studies analyzed in the meta-analysis (18, 19, 35); (Supplementary Table S2). Besides, some other studies provided the baseline CRP levels (26–29, 31, 33); (Supplementary Table S2), showing a significant elevation. Future studies could set upregulated level of CRP as one of the inclusion criteria to better guide JAK inhibitors usage.

Subgroup analysis according to baseline score on the NIAID ordinal scale showed that patients requiring supplemental oxygen, high-flow oxygen, or non-invasive ventilation benefited most from the baricitinib treatment. A previous study (5) displayed that serum levels of IL-6, IL-10, and TNF-α increase with disease severity in patients with COVID-19. As a result, compared with patients not requiring supplemental oxygen at baseline, patients above undergo more severe systematic hyperinflammation and thus are more likely to benefit from JAK inhibitor treatment. A similar trend was observed in other immunomodulatory drugs, such as corticosteroids and IL-6 receptor antagonists. For example, hospitalized patients not receiving supplementary oxygen do not benefit from corticosteroids (41). As for IL-6 receptor antagonists, the WHO meta-analysis that included 9,835 patients from 21 trials demonstrated that mortality decrease was most evident among those receiving respiratory support with oxygen by nasal cannula, face mask, high-flow nasal oxygen, or non-invasive ventilation, similar as baricitinib (42, 43). As for patients requiring IMV or ECMO, baricitinib exhibited no significant impact on mortality, similar to IL-6 antagonists (42). On the one hand, these patients experience more severe hyperinflammation. On the other hand, their alveolar damage and vascular injury might be too severe to be rescued (44). Which aspect will dominate and what is the efficacy of baricitinib in these patients need more evidence to determine.

The efficacy of corticosteroids has been well-recognized (8) and they were included in multiple clinical guidelines for COVID-19 across the world (45–47). Whether corticosteroids could be replaced by JAK inhibitors is unknown. One RCT named ACTT-4 (48), which planned to compare the efficacy of baricitinib plus remdesivir combination treatment with that of dexamethasone plus remdesivir combination treatment, closed the enrollment after interim efficacy analysis, because these two arms were very unlikely to have significantly different efficacy in hospitalized adult patients with COVID-19 in terms of the incidence of IMV or death (composite outcome). Moreover, one observational study of ruxolitinib (33) compared mortality between the ruxolitinib treatment group and the dexamethasone treatment group, demonstrating that they were comparable in terms of mortality. The evidence up to now suggested that JAK inhibitors might be an alternative therapy for patients with contradictions of corticosteroids, though could not replace them considering availability and pharmaceutical economics.

In the WHO meta-analysis (42), the effect of IL-6 receptor antagonists to decrease all-cause mortality by day 28 was only observed in patients with concomitant corticosteroids treatment. Considering the similar anti-inflammation mechanisms of IL-6 receptor antagonists and JAK inhibitors, we hoped to conduct subgroup analysis according to whether having concomitant corticosteroids treatment to better guide the usage of JAK inhibitors, but was not feasible as only COV-BARRIER and its addendum trial (18, 19) provided related data. Due to the limited sample size of no corticosteroids group, new evidence is needed for more solid conclusions.

Baricitinib, ruxolitinib, and tofacitinib are all oral JAK inhibitors, while nezulcitinib is an inhaled one. The drug was designated as a potential treatment for acute lung injury associated with COVID-19 initially in 2020 (49). Data included in this systematic review were generated from part 1 of the phase II trial (37), which aimed to explore the optimal dose with a small sample size (*n* = 25). As an inhaled drug, nezulcitinib might have a relatively low level of systemic absorption compared with high local concentration in the lung (50), possibly leading to altered efficacy, incidence of systematic adverse events, and optimal patient population. All in all, the ongoing second part of this phase II trial launched in a larger sample (about 200) would provide more clues for these questions (37).

Several systematic reviews and meta-analyses have been published related to JAK inhibitor treatment for patients with COVID-19 (51–53). Our study has some strengths compared with them. First, our analysis was designed as a living one. Thus, with new evidence publishing, we could update our analysis in time to give the newest indications for clinical practice. Next, more types of JAK inhibitors were included in our analysis, with tofacitinib and inhaled nezulcitinib, providing a more comprehensive understanding of JAK inhibitors. Moreover, the quality of both RCTs and observational studies evaluated here was all relatively high, and our major conclusions were generated from the RCTs, making them less likely to have bias. Finally, subgroup analysis was conducted in our study, which facilitates seeking for the optimal patient population.

Still, our study has some limitations. The population included here was mostly hospitalized adult patients with COVID-19. Safety and efficacy in some special populations, such as pediatric patients, need further studies. Then, intervention across studies were heterogenous, from the type of JAK inhibitors, time to start treatment, dose, and frequency, to duration, which obscured guidance for clinical practice. Furthermore, the follow-up time of included studies was relatively short, mostly about 28 days. Therefore, the long-term effects of JAK inhibitors remain unknown. In addition, our conclusions in this version were mainly generated for baricitinib. Whether the conclusions could be generalized to other types of JAK inhibitors awaits more data.

Based on the results of the ACTT-2 trial (17) and the COV-BARRIER trial (18), U.S. FDA issued an Emergency Use Authorization (EUA) for emergency use of baricitinib in patients requiring supplemental oxygen, non-invasive mechanical ventilation, IMV, or ECMO (54). Moreover, COVID-19 treatment guidelines developed by the National Institutes of Health also included baricitinib and tofacitinib (45). With clinical studies getting finished and published, we believe that baricitinib and other JAK inhibitors would be included in more guidelines, and the optimal patient population, regimen, and combination therapy would also be clarified. We will keep tracking new evidence and update this study when evidence potentially changing previous conclusions is published.

## Conclusion

Meta-analysis on RCTs indicated that baricitinib could decrease day 28 mortality in hospitalized adult patients with COVID-19 and patients requiring supplemental oxygen or high-flow oxygen/non-invasive ventilation at baseline benefited most. Meanwhile, it would not increase the incidence of adverse events, serious adverse events, or infection or secondary infection. The efficacy and safety of the remaining three JAK inhibitors, ruxolitinib, tofacitinib, and nezulcitinib, await more evidence.

## Data Availability Statement

The original contributions presented in the study are included in the article/Supplementary Material, further inquiries can be directed to the corresponding author.

## Author Contributions

BC: conceived the study. XZ and LS: designed the protocol, performed article screening, data extraction, and statistical analysis. XZ: wrote the first manuscript draft. LS, BC, GF, XG, JX, YW, and LH: provided critical revisions. All authors contributed to the article and approved the submitted version.

## Funding

This work was supported by the Chinese Academy of Medical Sciences Innovation Fund for Medical Sciences [2020-I2M-CoV19-005 and 2018-I2M-1-003] and the National Natural Science Foundation of China [82041011].

## Conflict of Interest

The authors declare that the research was conducted in the absence of any commercial or financial relationships that could be construed as a potential conflict of interest.

## Publisher's Note

All claims expressed in this article are solely those of the authors and do not necessarily represent those of their affiliated organizations, or those of the publisher, the editors and the reviewers. Any product that may be evaluated in this article, or claim that may be made by its manufacturer, is not guaranteed or endorsed by the publisher.
