# Supplementary material for: The Efficacy and Safety of Janus Kinase Inhibitors for Patients With COVID-19: A Living Systematic Review and Meta-Analysis
Source: Front Med (Lausanne). 2022 Jan 27;8:800492. doi: 10.3389/fmed.2021.800492 (PMC8828941; doi:10.3389/fmed.2021.800492)
Supplement: Supplementary file 1 [file Data_Sheet_1.pdf]

Supplement material

Manuscript title: The Efficacy and Safety of Janus Kinase Inhibitors for Patients With COVID-19: A Living Systematic Review and Meta-Analysis.

Authors: Xueyang Zhang, Lianhan Shang, Guohui Fan, Xiaoying Gu, Jiuyang Xu, Yeming Wang, Lixue Huang, Bin Cao.

Contents

Table S1. Risk of bias assessment of included observational studies 2

Table S2. Baseline C-reactive protein level of included studies 3

Figure S1. Risk of bias assessment of included randomized controlled trials 4

Figure S2. Forest plots for efficacy outcomes with baricitinib vs. control 5

Figure S3. Forest plots for efficacy and safety outcomes with tofacitinib vs. control 6

Figure S4. Forest plots for efficacy and safety outcomes with ruxolitinib vs. control 8

Figure S5. Forest plots for efficacy and safety outcomes with nezulcitinib vs. control 9

Figure S6. Forest plots for efficacy and safety outcomes with Janus kinase inhibitor vs. control 10

Figure S7. Sensitivity analysis for efficacy and safety outcomes with Janus kinase inhibitor vs. control 12

Figure S8. Funnel plots for efficacy and safety outcomes with Janus kinase inhibitor vs. control 14

Appendix 1. PRISMA check-list 15

Appendix 2. PROSPERO registration 18

Appendix 3. Search strategies 23

Table S1. Risk of bias assessment of included observational studies using Newcastle-Ottawa scale.

First author	JAK inhibitor	Total	Selection	Comparability	Exposure/ Outcome
Bronte, V.	Baricitinib	7/9	4/4	1/2	2/3
Rosas, J.	Baricitinib	8/9	4/4	1/2	3/3
Stebbing, J.	Baricitinib	7/9	3/4	1/2	3/3
Pérez-Alba, E.	Baricitinib	6/9	3/4	0/2	3/3
Abizanda, P.	Baricitinib	8/9	4/4	1/2	3/3
Masiá, M.	Baricitinib	8/9	4/4	1/2	3/3
Stanevich, O.V. Pre-print	Ruxolitinib	8/9	4/4	1/2	3/3
Maslennikov, R.	Tofacitinib	7/9	3/4	1/2	3/3
Singh, P.K.	Tofacitinib	6/9	2/4	1/2	3/3

Table S2. Baseline C-reactive protein level of included studies.

First author	JAK inhibitor	Study design	Baseline CRP (mg/L)
Kalil, A.C.	Baricitinib	RCT	NA
Marconi, V.C.	Baricitinib	RCT	One of the inclusion criteria: patients with at least one elevated inflammatory marker (C-reactive protein, D-dimer, lactate dehydrogenase, or ferritin).
Ely, E.W.<br>Pre-print	Baricitinib	RCT	One of the inclusion criteria: patients with at least one elevated inflammatory marker (C-reactive protein, D-dimer, lactate dehydrogenase, or ferritin).<br>Intervention group (median): 124.9.<br>Control group (median): 109.5.
Bronte, V.	Baricitinib	Observational study	Intervention group (median [IQR]): 53.15 (43.08-77.63).<br>Control group (median [IQR]): 64.5 (37-130.3).
Rosas, J.	Baricitinib	Observational study	Baricitinib group (mean [SD]): 166 (102).<br>Baricitinib plus tocilizumab group (mean [SD]): 179 (115).<br>No baricitinib or tocilizumab group (mean [SD]): 125 (83).<br>Tocilizumab group (mean [SD]): 157 (76).
Stebbing, J.	Baricitinib	Observational study	Intervention group (median [IQR]): 91.2 (59-165).<br>Control group (median [IQR]): 43 (15-52).
Pérez-Alba, E.	Baricitinib	Observational study	Intervention group (mean [SD]): 166 (95).<br>Control group (mean [SD]): 170 (99).
Abizanda, P.	Baricitinib	Observational study	NA
Masiá, M.	Baricitinib	Observational study	Intervention group (median [IQR]): 33 (6-105).<br>Control group (median [IQR]): 81 (50-129).
Cao, Y.	Ruxolitinib	RCT	NA
Stanevich, O.V.<br>Pre-print	Ruxolitinib	Observational study	Intervention group (mean [SD]): 97.4 (71.9)<br>Control group (mean [SD]): 99.5 (84.7).
Guimarães, P.O.	Tofacitinib	RCT	NA
Maslennikov, R.	Tofacitinib	Observational study	One of the inclusion criteria: patients with CRP between 60 and 150 mg/L.<br>Intervention group (median [IQR]): 95 (73-140).<br>Control group (median [IQR]): 110 (94-131).
Singh, P.K.	Tofacitinib	Observational study	NA
Singh, D.	Nezulcitinib	RCT	NA

Abbreviations: CRP, C-reactive protein; IQR, interquartile range; JAK, Janus kinase; NA, not available; SD, standard deviation.

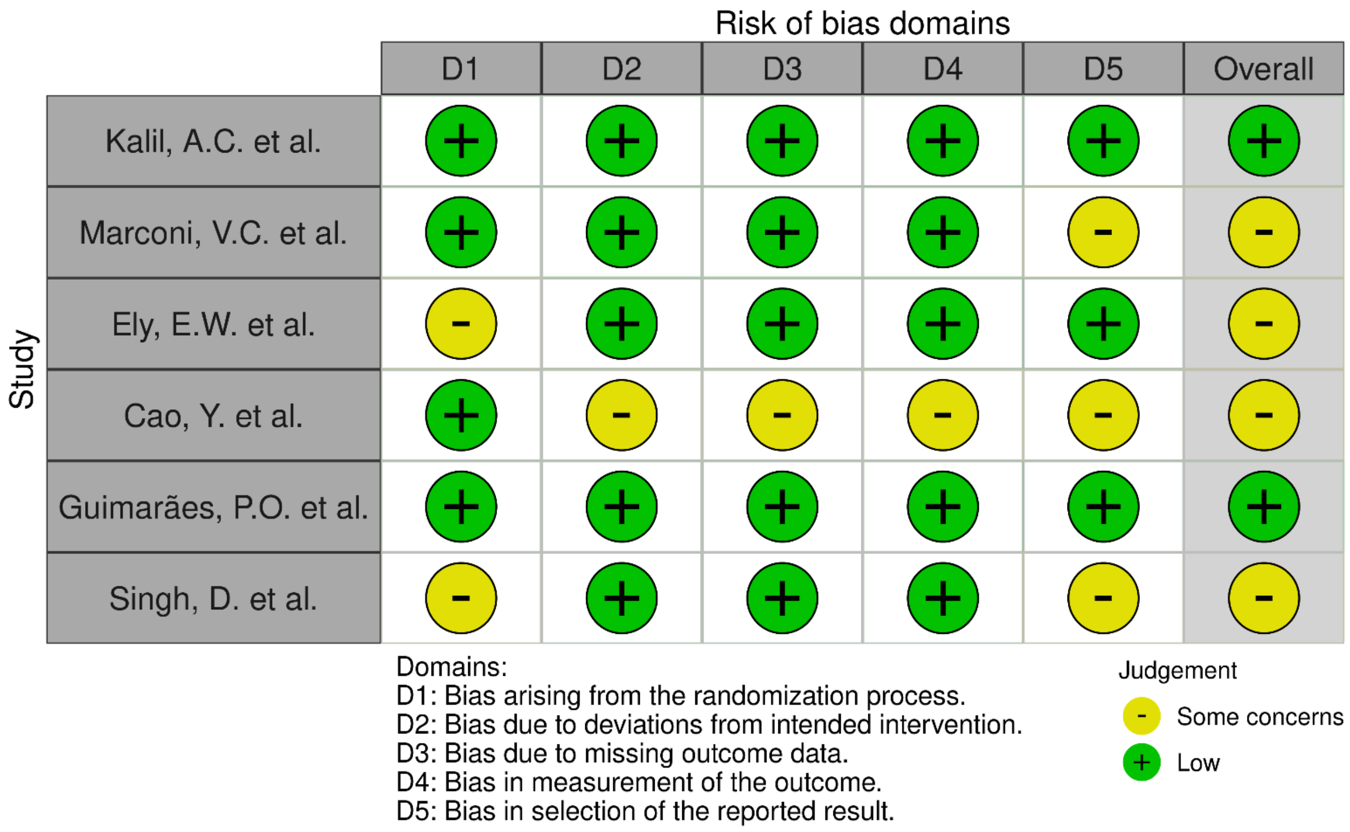

Figure S1. Risk of bias assessment of included randomized controlled trials using Cochrane risk-of-bias 2.0 tool.

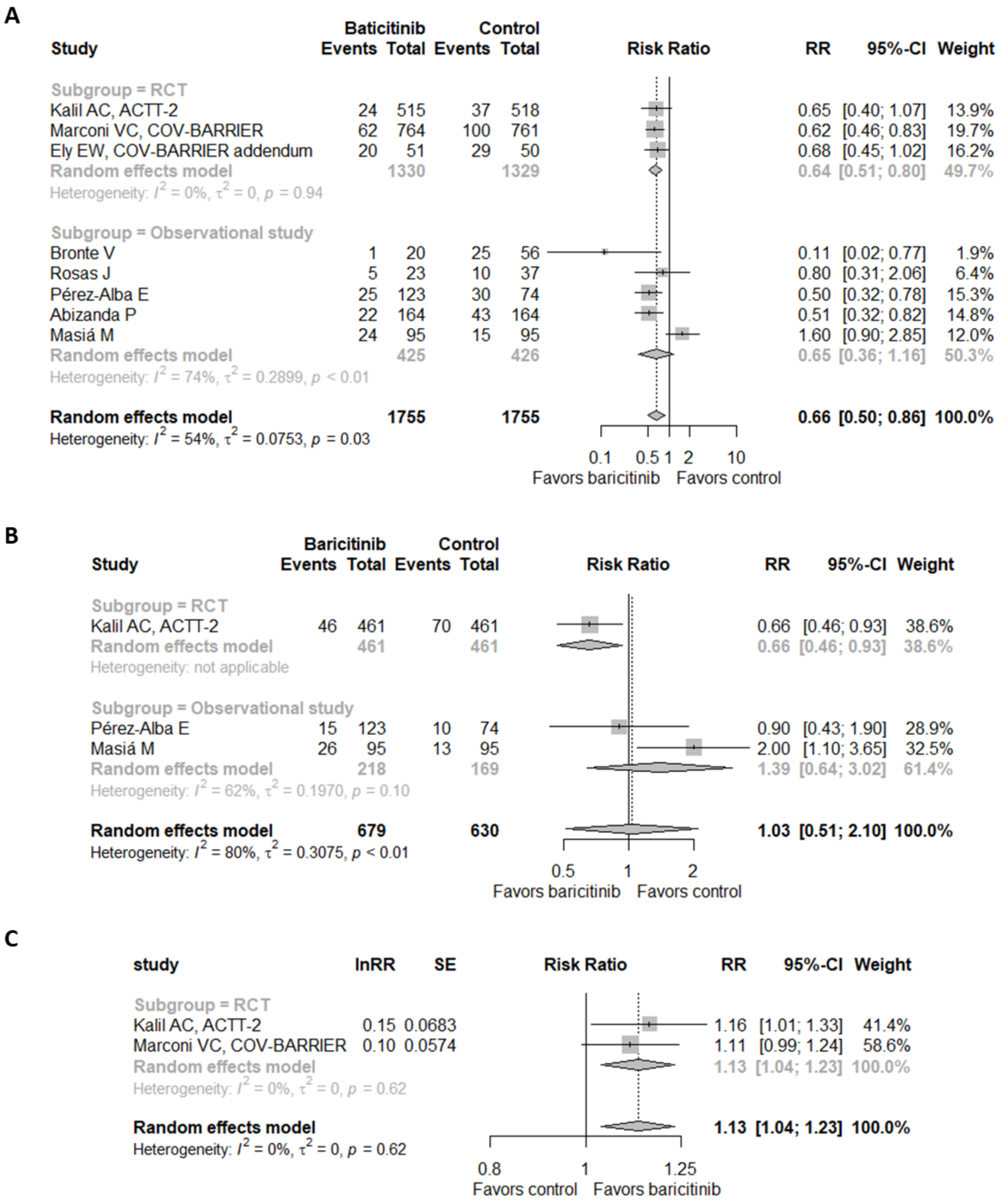

Figure S2. Forest plots for efficacy outcomes with baricitinib vs. control in randomized controlled trials and observational studies. A: mortality. B: incidence of invasive mechanical ventilation. C: time to recovery. RR, risk ratio; CI, confidence interval; SE, standard error.

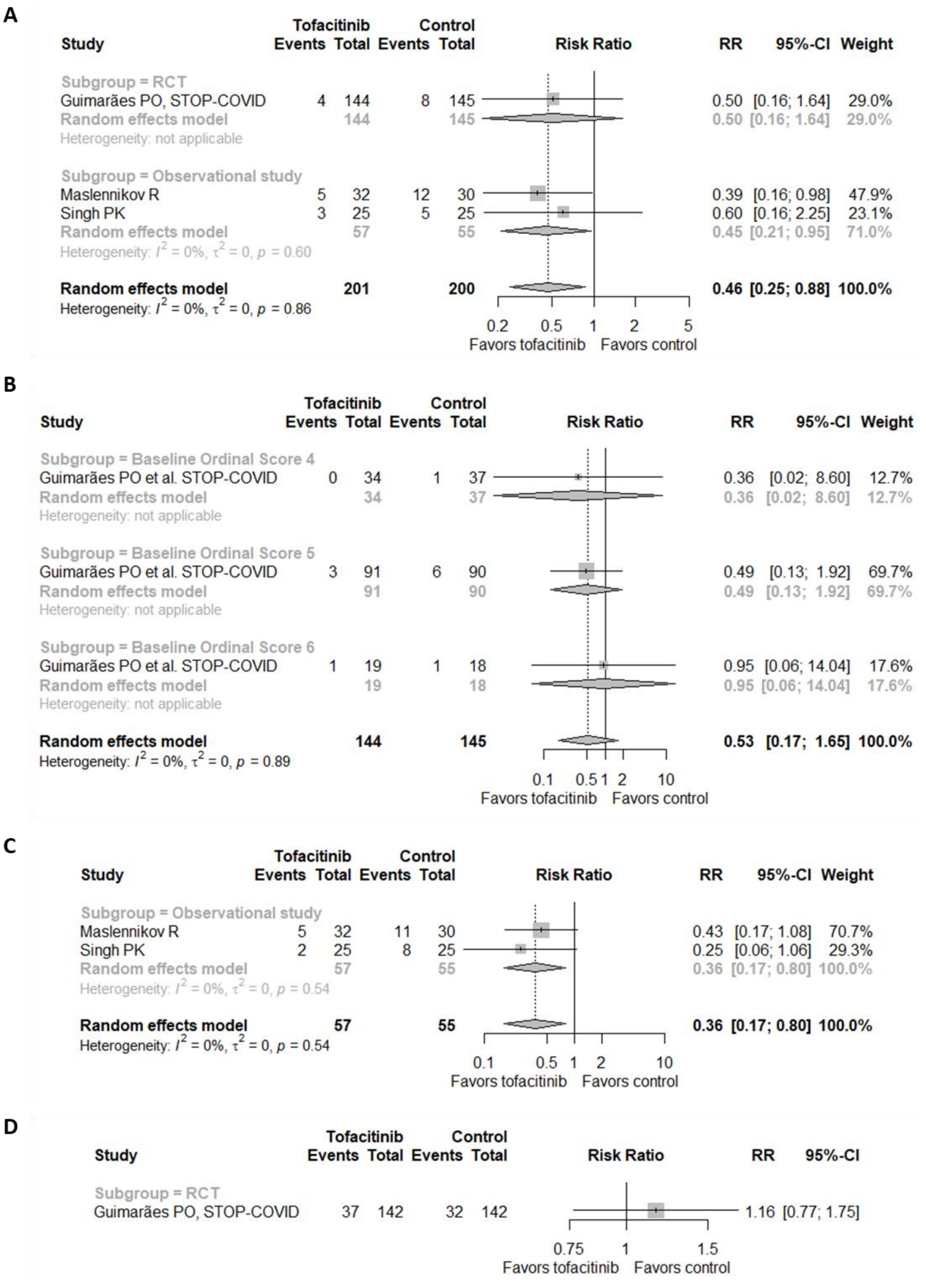
A
Study
Tofacitinib
Events Total
Control
Events Total
Risk Ratio
RR 95%-CI Weight
Subgroup = RCT
Guimarães PO, STOP-COVID 4 144 8 145 0.50 [0.16; 1.64] 29.0%
Random effects model 144 145 0.50 [0.16; 1.64] 29.0%
Heterogeneity: not applicable
Subgroup = Observational study
Maslennikov R 5 32 12 30 0.39 [0.16; 0.98] 47.9%
Singh PK 3 25 5 25 0.60 [0.16; 2.25] 23.1%
Random effects model 57 55 0.45 [0.21; 0.95] 71.0%
Heterogeneity: I^2 = 0%, τ^2 = 0, p = 0.60
Random effects model 201 200 0.46 [0.25; 0.88] 100.0%
Heterogeneity: I^2 = 0%, τ^2 = 0, p = 0.86
0.2 0.5 1 2 5
Favors tofacitinib Favors control
B
Study
Tofacitinib
Events Total
Control
Events Total
Risk Ratio
RR 95%-CI Weight
Subgroup = Baseline Ordinal Score 4
Guimarães PO et al. STOP-COVID 0 34 1 37 0.36 [0.02; 8.60] 12.7%
Random effects model 34 37 0.36 [0.02; 8.60] 12.7%
Heterogeneity: not applicable
Subgroup = Baseline Ordinal Score 5
Guimarães PO et al. STOP-COVID 3 91 6 90 0.49 [0.13; 1.92] 69.7%
Random effects model 91 90 0.49 [0.13; 1.92] 69.7%
Heterogeneity: not applicable
Subgroup = Baseline Ordinal Score 6
Guimarães PO et al. STOP-COVID 1 19 1 18 0.95 [0.06; 14.04] 17.6%
Random effects model 19 18 0.95 [0.06; 14.04] 17.6%
Heterogeneity: not applicable
Random effects model 144 145 0.53 [0.17; 1.65] 100.0%
Heterogeneity: I^2 = 0%, τ^2 = 0, p = 0.89
0.1 0.5 1 2 10
Favors tofacitinib Favors control
C
Study
Tofacitinib
Events Total
Control
Events Total
Risk Ratio
RR 95%-CI Weight
Subgroup = Observational study
Maslennikov R 5 32 11 30 0.43 [0.17; 1.08] 70.7%
Singh PK 2 25 8 25 0.25 [0.06; 1.06] 29.3%
Random effects model 57 55 0.36 [0.17; 0.80] 100.0%
Heterogeneity: I^2 = 0%, τ^2 = 0, p = 0.54
Random effects model 57 55 0.36 [0.17; 0.80] 100.0%
Heterogeneity: I^2 = 0%, τ^2 = 0, p = 0.54
0.1 0.5 1 2 10
Favors tofacitinib Favors control
D
Study
Tofacitinib
Events Total
Control
Events Total
Risk Ratio
RR 95%-CI
Subgroup = RCT
Guimarães PO, STOP-COVID 37 142 32 142 1.16 [0.77; 1.75]
0.75 1 1.5
Favors tofacitinib Favors control

E

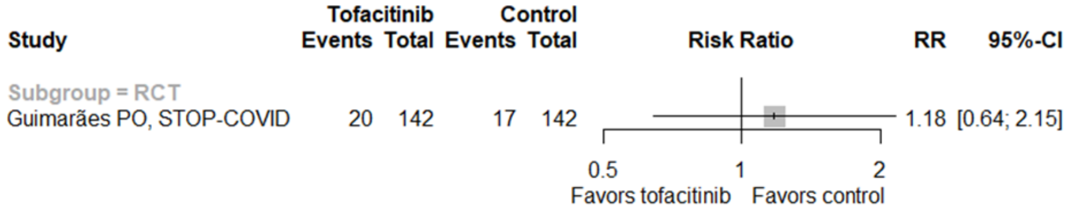

F

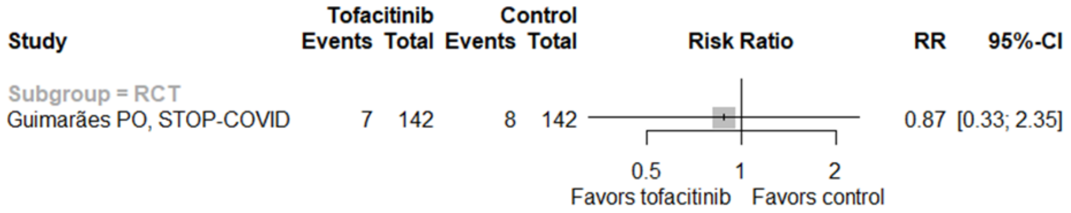

Figure S3. Forest plots for efficacy and safety outcomes with tofacitinib vs. control in randomized controlled trials and observational studies. A: mortality. B. subgroup analysis of mortality according to the baseline National Institute of Allergy and Infectious Diseases ordinal scale score. C: incidence of invasive mechanical ventilation. D:adverse events. E: serious adverse events. F: infection or secondary infection. RR, risk ratio; CI, confidence interval.

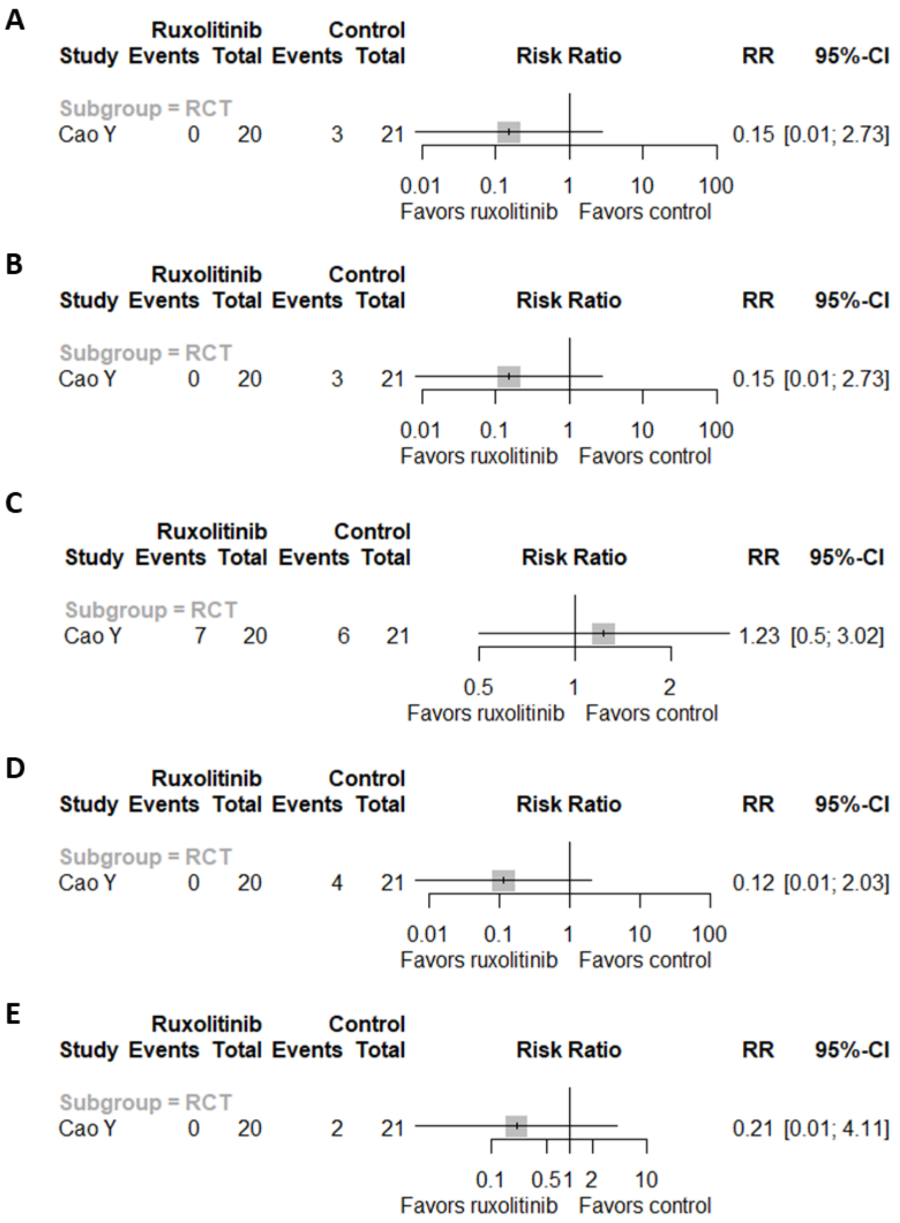

Figure S4. Forest plots for efficacy and safety outcomes with ruxolitinib vs. control in randomized controlled trials and observational studies. A: mortality. B: incidence of invasive mechanical ventilation. C: adverse events. D: serious adverse events. E: infection or secondary infection. RR, risk ratio; CI, confidence interval.

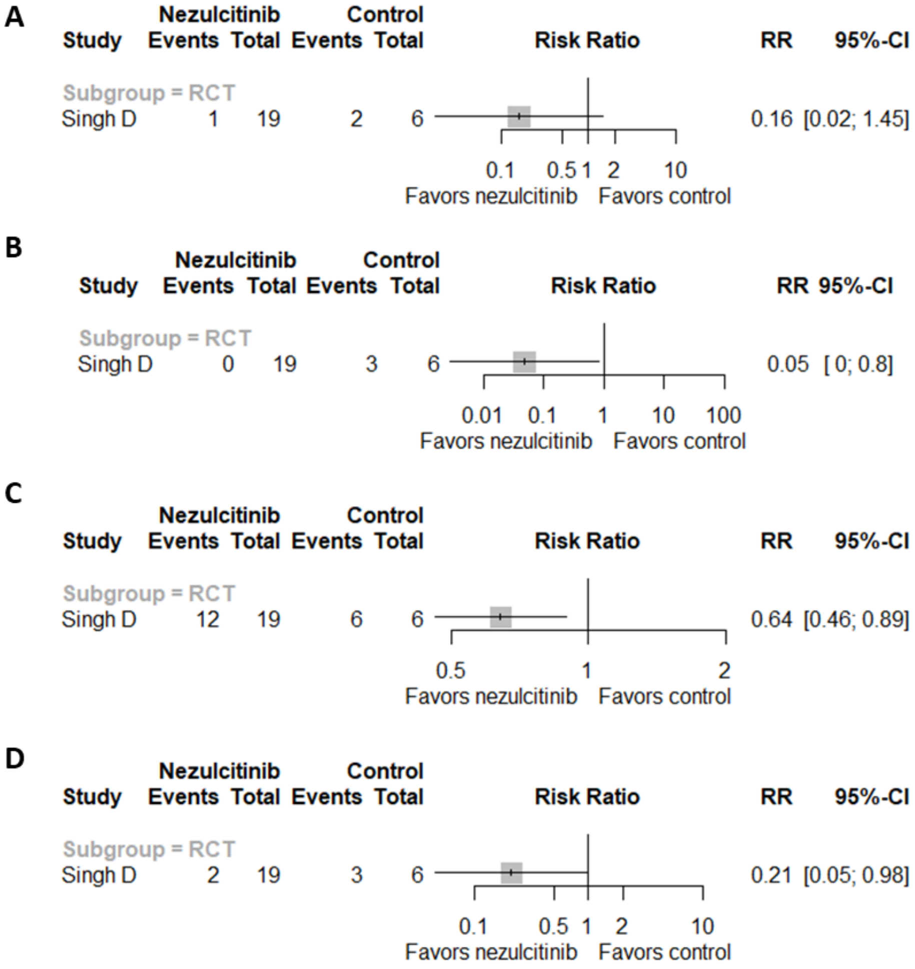

Figure S5. Forest plots for efficacy and safety outcomes with nezulcitinib vs. control in randomized controlled trials and observational studies. A: mortality. B: incidence of invasive mechanical ventilation. C: adverse events. D: serious adverse events. RR, risk ratio; CI, confidence interval.

A

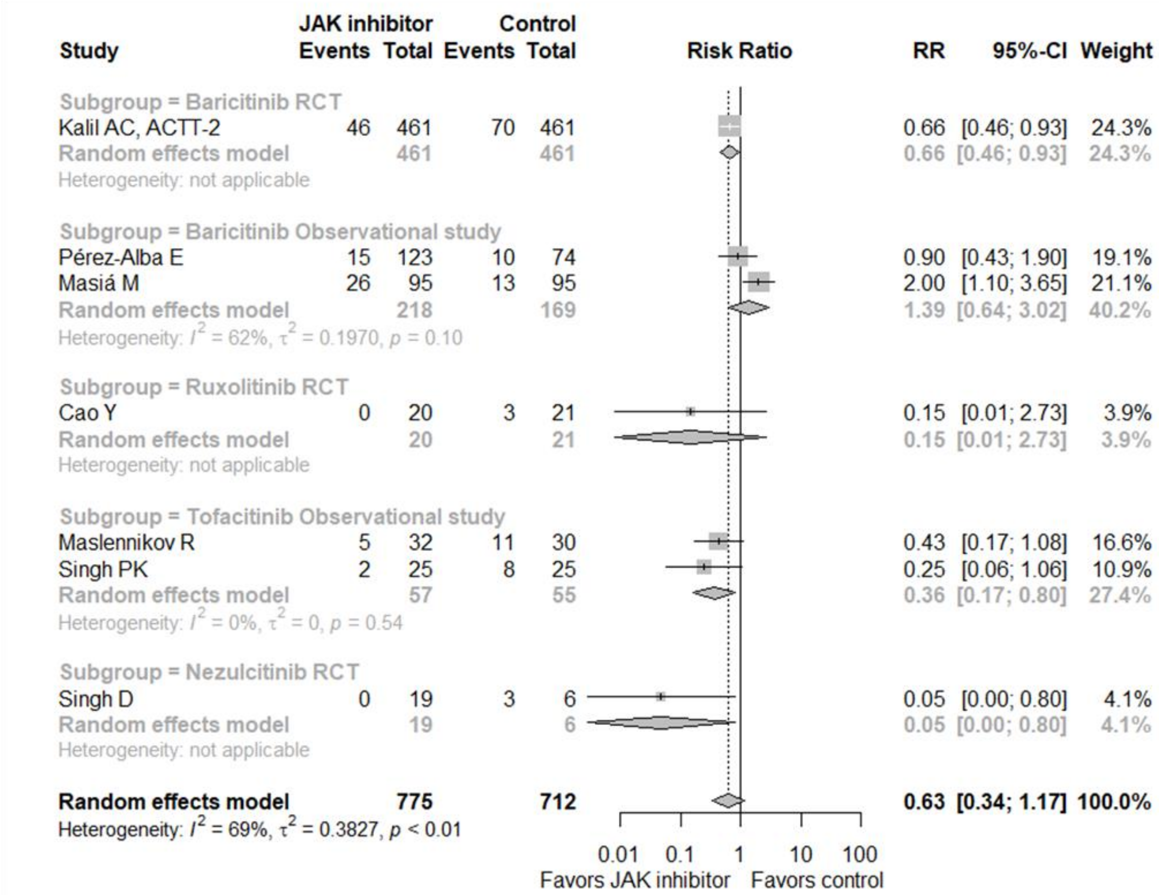
JAK inhibitor
Control
Study
Events Total Events Total
Risk Ratio
RR 95%-CI Weight
Subgroup = Baricitinib RCT
Kalil AC, ACTT-2 46 461 70 461 0.66 [0.46; 0.93] 24.3%
Random effects model 461 461 0.66 [0.46; 0.93] 24.3%
Heterogeneity: not applicable
Subgroup = Baricitinib Observational study
Pérez-Alba E 15 123 10 74 0.90 [0.43; 1.90] 19.1%
Masiá M 26 95 13 95 2.00 [1.10; 3.65] 21.1%
Random effects model 218 169 1.39 [0.64; 3.02] 40.2%
Heterogeneity: I^2 = 62%, τ^2 = 0.1970, p = 0.10
Subgroup = Ruxolitinib RCT
Cao Y 0 20 3 21 0.15 [0.01; 2.73] 3.9%
Random effects model 20 21 0.15 [0.01; 2.73] 3.9%
Heterogeneity: not applicable
Subgroup = Tofacitinib Observational study
Maslennikov R 5 32 11 30 0.43 [0.17; 1.08] 16.6%
Singh PK 2 25 8 25 0.25 [0.06; 1.06] 10.9%
Random effects model 57 55 0.36 [0.17; 0.80] 27.4%
Heterogeneity: I^2 = 0%, τ^2 = 0, p = 0.54
Subgroup = Nezulcitinib RCT
Singh D 0 19 3 6 0.05 [0.00; 0.80] 4.1%
Random effects model 19 6 0.05 [0.00; 0.80] 4.1%
Heterogeneity: not applicable
Random effects model 775 712 0.63 [0.34; 1.17] 100.0%
Heterogeneity: I^2 = 69%, τ^2 = 0.3827, p < 0.01
0.01 0.1 1 10 100
Favors JAK inhibitor Favors control

B

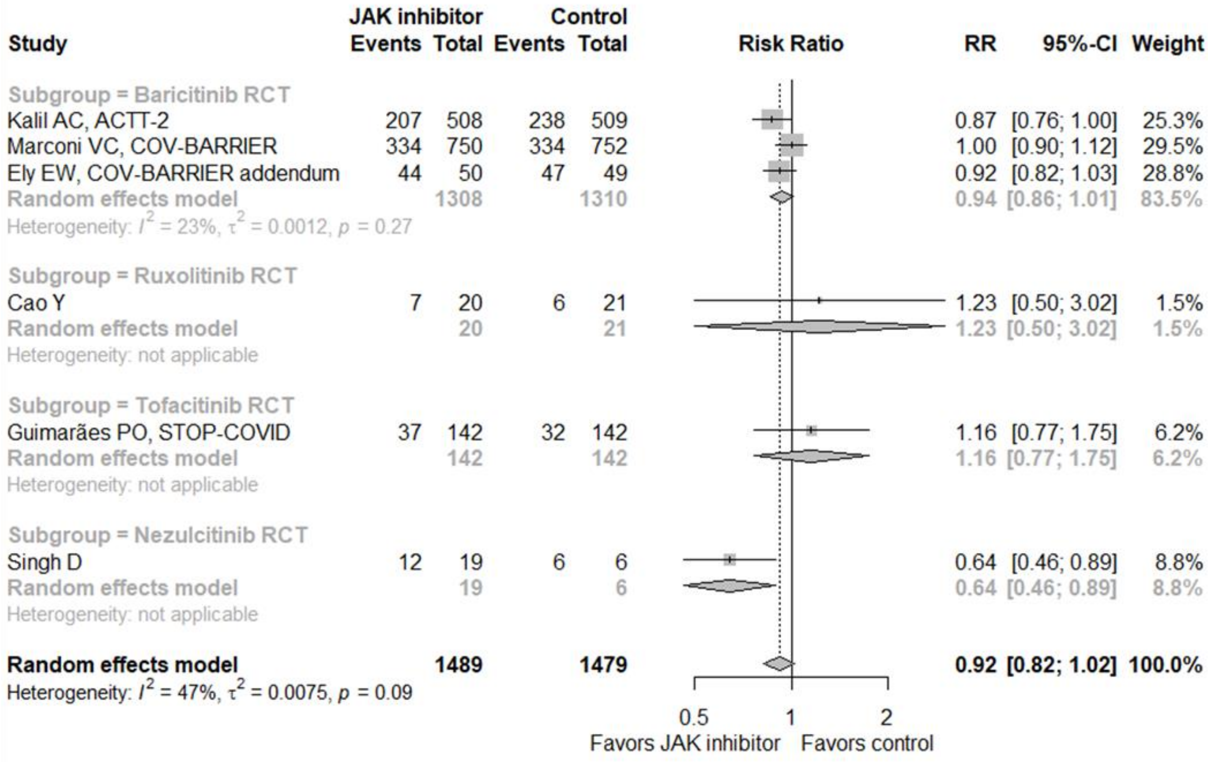
JAK inhibitor
Control
Study
Events Total Events Total
Risk Ratio
RR 95%-CI Weight
Subgroup = Baricitinib RCT
Kalil AC, ACTT-2 207 508 238 509 0.87 [0.76; 1.00] 25.3%
Marconi VC, COV-BARRIER 334 750 334 752 1.00 [0.90; 1.12] 29.5%
Ely EW, COV-BARRIER addendum 44 50 47 49 0.92 [0.82; 1.03] 28.8%
Random effects model 1308 1310 0.94 [0.86; 1.01] 83.5%
Heterogeneity: I^2 = 23%, τ^2 = 0.0012, p = 0.27
Subgroup = Ruxolitinib RCT
Cao Y 7 20 6 21 1.23 [0.50; 3.02] 1.5%
Random effects model 20 21 1.23 [0.50; 3.02] 1.5%
Heterogeneity: not applicable
Subgroup = Tofacitinib RCT
Guimarães PO, STOP-COVID 37 142 32 142 1.16 [0.77; 1.75] 6.2%
Random effects model 142 142 1.16 [0.77; 1.75] 6.2%
Heterogeneity: not applicable
Subgroup = Nezulcitinib RCT
Singh D 12 19 6 6 0.64 [0.46; 0.89] 8.8%
Random effects model 19 6 0.64 [0.46; 0.89] 8.8%
Heterogeneity: not applicable
Random effects model 1489 1479 0.92 [0.82; 1.02] 100.0%
Heterogeneity: I^2 = 47%, τ^2 = 0.0075, p = 0.09
0.5 1 2
Favors JAK inhibitor Favors control

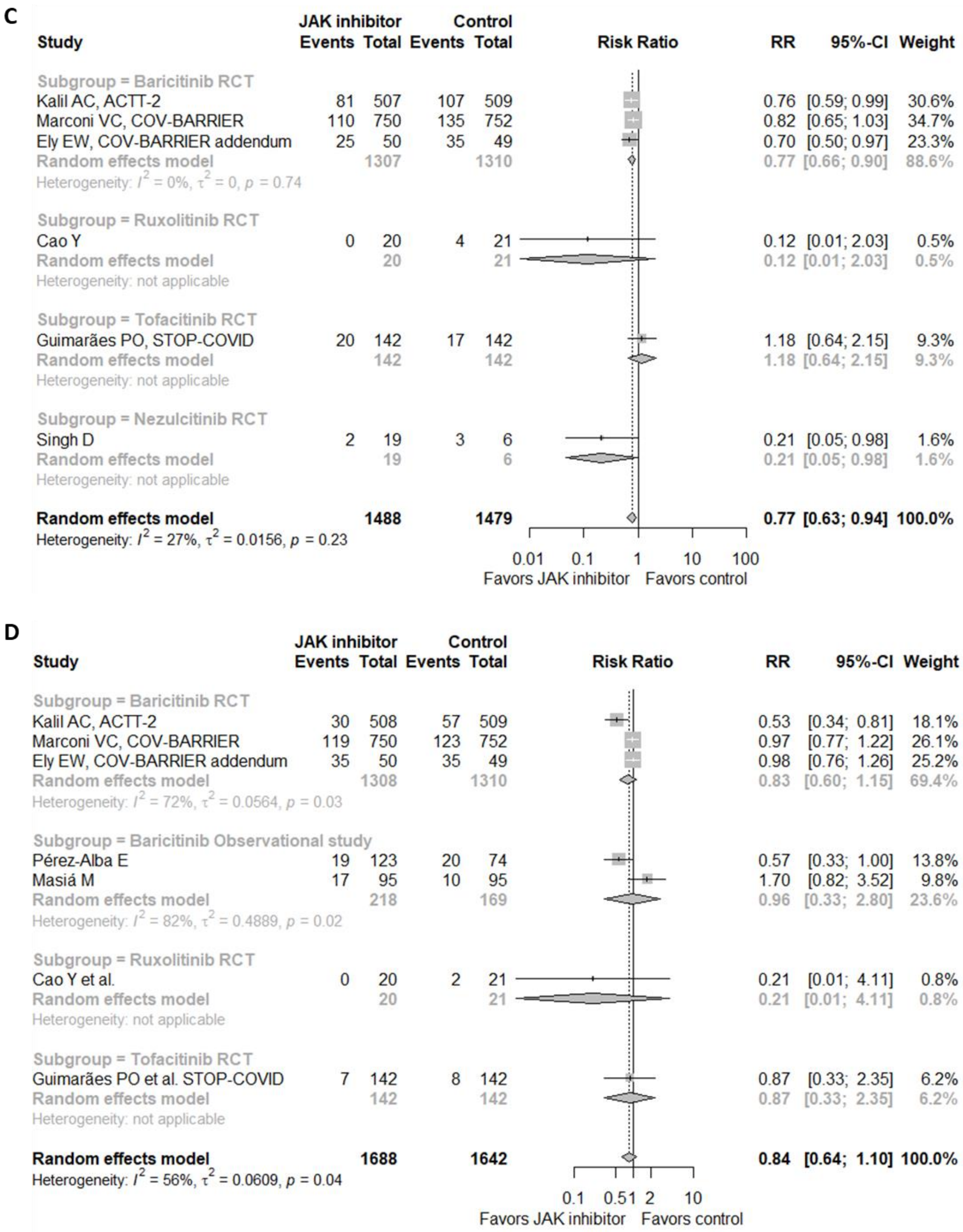

Figure S6. Forest plots for efficacy and safety outcomes with Janus kinase inhibitor vs. control in randomized controlled trials and observational studies. A: incidence of invasive mechanical ventilation. B: adverse events. C: serious adverse events. D: infection or secondary infection. JAK, Janus kinase; RR, risk ratio; CI, confidence interval.

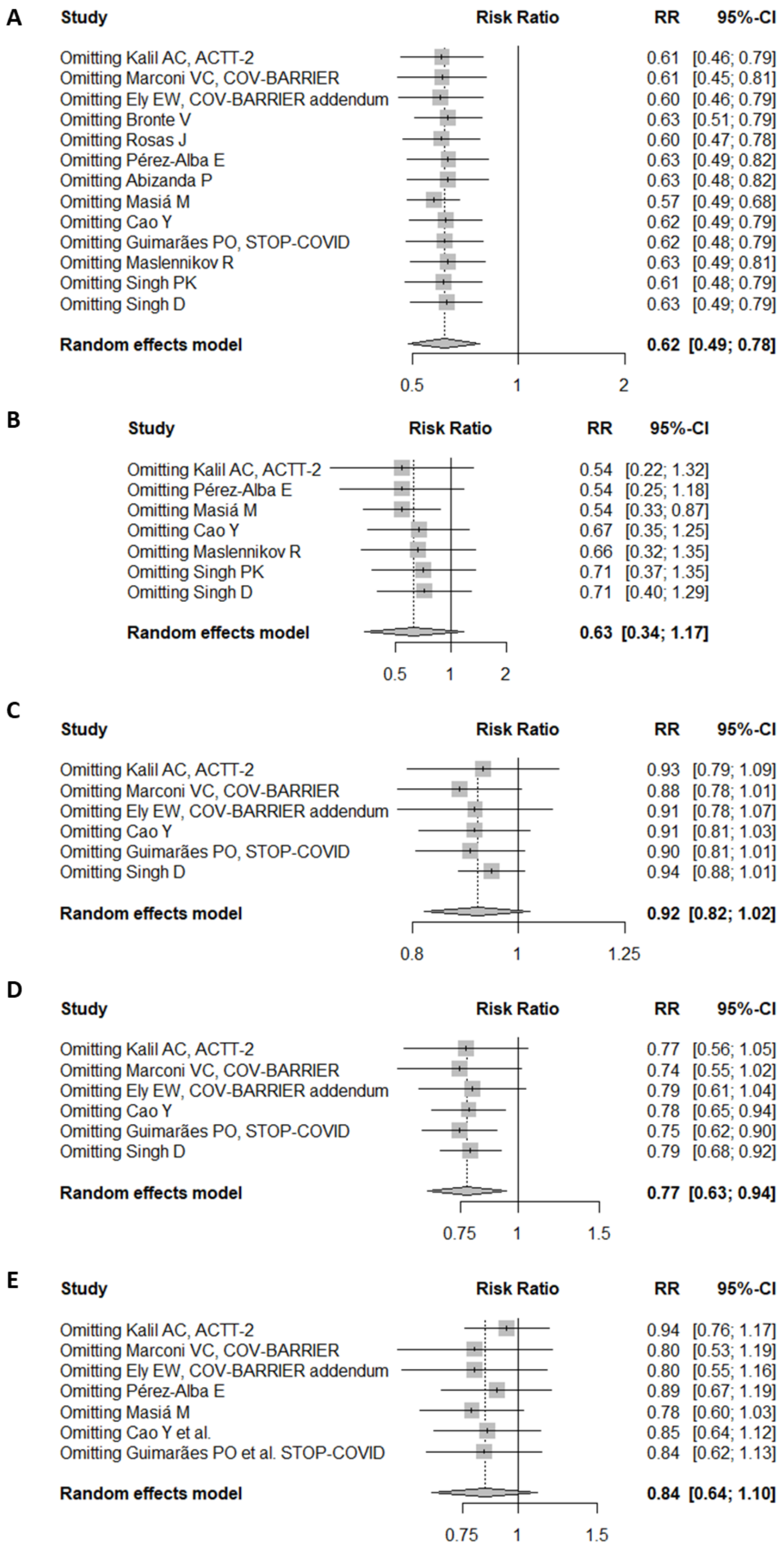
A
Study
Risk Ratio
RR
95%-CI
Omitting Kalil AC, ACTT-2 0.61 [0.46; 0.79]
Omitting Marconi VC, COV-BARRIER 0.61 [0.45; 0.81]
Omitting Ely EW, COV-BARRIER addendum 0.60 [0.46; 0.79]
Omitting Bronte V 0.63 [0.51; 0.79]
Omitting Rosas J 0.60 [0.47; 0.78]
Omitting Pérez-Alba E 0.63 [0.49; 0.82]
Omitting Abizanda P 0.63 [0.48; 0.82]
Omitting Masiá M 0.57 [0.49; 0.68]
Omitting Cao Y 0.62 [0.49; 0.79]
Omitting Guimarães PO, STOP-COVID 0.62 [0.48; 0.79]
Omitting Maslennikov R 0.63 [0.49; 0.81]
Omitting Singh PK 0.61 [0.48; 0.79]
Omitting Singh D 0.63 [0.49; 0.79]
Random effects model 0.62 [0.49; 0.78]
0.5
1
2
B
Study
Risk Ratio
RR
95%-CI
Omitting Kalil AC, ACTT-2 0.54 [0.22; 1.32]
Omitting Pérez-Alba E 0.54 [0.25; 1.18]
Omitting Masiá M 0.54 [0.33; 0.87]
Omitting Cao Y 0.67 [0.35; 1.25]
Omitting Maslennikov R 0.66 [0.32; 1.35]
Omitting Singh PK 0.71 [0.37; 1.35]
Omitting Singh D 0.71 [0.40; 1.29]
Random effects model 0.63 [0.34; 1.17]
0.5
1
2
C
Study
Risk Ratio
RR
95%-CI
Omitting Kalil AC, ACTT-2 0.93 [0.79; 1.09]
Omitting Marconi VC, COV-BARRIER 0.88 [0.78; 1.01]
Omitting Ely EW, COV-BARRIER addendum 0.91 [0.78; 1.07]
Omitting Cao Y 0.91 [0.81; 1.03]
Omitting Guimarães PO, STOP-COVID 0.90 [0.81; 1.01]
Omitting Singh D 0.94 [0.88; 1.01]
Random effects model 0.92 [0.82; 1.02]
0.8
1
1.25
D
Study
Risk Ratio
RR
95%-CI
Omitting Kalil AC, ACTT-2 0.77 [0.56; 1.05]
Omitting Marconi VC, COV-BARRIER 0.74 [0.55; 1.02]
Omitting Ely EW, COV-BARRIER addendum 0.79 [0.61; 1.04]
Omitting Cao Y 0.78 [0.65; 0.94]
Omitting Guimarães PO, STOP-COVID 0.75 [0.62; 0.90]
Omitting Singh D 0.79 [0.68; 0.92]
Random effects model 0.77 [0.63; 0.94]
0.75
1
1.5
E
Study
Risk Ratio
RR
95%-CI
Omitting Kalil AC, ACTT-2 0.94 [0.76; 1.17]
Omitting Marconi VC, COV-BARRIER 0.80 [0.53; 1.19]
Omitting Ely EW, COV-BARRIER addendum 0.80 [0.55; 1.16]
Omitting Pérez-Alba E 0.89 [0.67; 1.19]
Omitting Masiá M 0.78 [0.60; 1.03]
Omitting Cao Y et al. 0.85 [0.64; 1.12]
Omitting Guimarães PO et al. STOP-COVID 0.84 [0.62; 1.13]
Random effects model 0.84 [0.64; 1.10]
0.75
1
1.5

Figure S7. Sensitivity analysis by omitting any single study for efficacy and safety outcomes with Janus kinase inhibitor vs. control in randomized controlled trials and observational studies. A: mortality. B: incidence of invasive mechanical ventilation. C: adverse events. D: serious adverse events. E: infection or secondary infection. RR, risk ratio; CI, confidence interval.

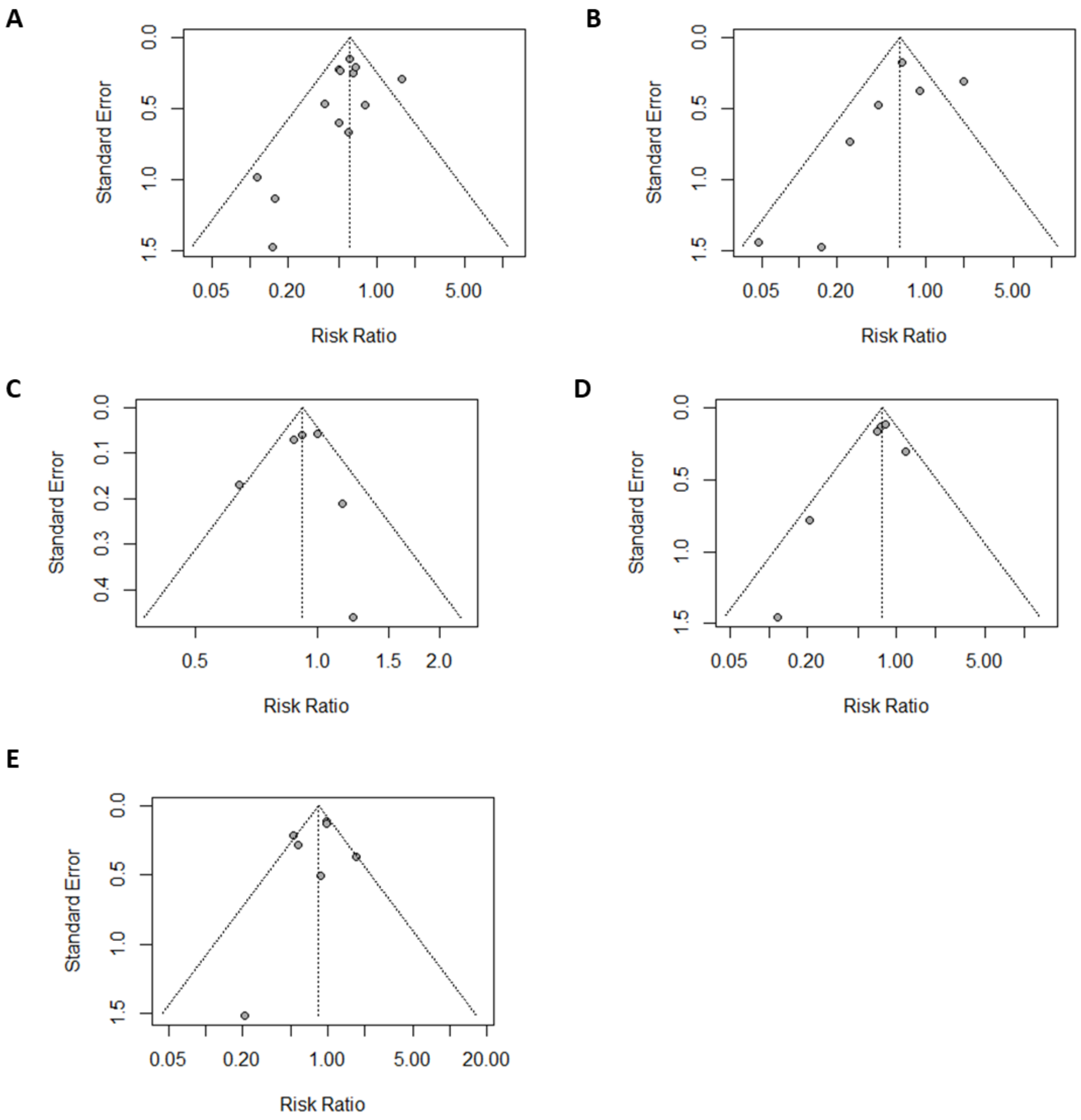

Figure S8. Funnel plots for efficacy and safety outcomes with Janus kinase inhibitor vs. control in randomized controlled trials and observational studies. A: mortality. B: incidence of invasive mechanical ventilation. C: adverse events. D: serious adverse events. E: infection or secondary infection.

Appendix 1. PRISMA check-list

Section/topic	#	Checklist item	Reported on page #
**TITLE**			
Title	1	Identify the report as a systematic review, meta-analysis, or both.	1
**ABSTRACT**			
Structured summary	2	Provide a structured summary including, as applicable: background; objectives; data sources; study eligibility criteria, participants, and interventions; study appraisal and synthesis methods; results; limitations; conclusions and implications of key findings; systematic review registration number.	1
**INTRODUCTION**			
Rationale	3	Describe the rationale for the review in the context of what is already known.	2
Objectives	4	Provide an explicit statement of questions being addressed with reference to participants, interventions, comparisons, outcomes, and study design (PICOS).	2
**METHODS**			
Protocol and registration	5	Indicate if a review protocol exists, if and where it can be accessed (e.g., Web address), and, if available, provide registration information including registration number.	2
Eligibility criteria	6	Specify study characteristics (e.g., PICOS, length of follow-up) and report characteristics (e.g., years considered, language, publication status) used as criteria for eligibility, giving rationale.	2
Information sources	7	Describe all information sources (e.g., databases with dates of coverage, contact with study authors to identify additional studies) in the search and date last searched.	2<br>Appendix 1
Search	8	Present full electronic search strategy for at least one database, including any limits used, such that it could be repeated.	Appendix 1
Study selection	9	State the process for selecting studies (i.e., screening, eligibility, included in systematic review, and, if applicable, included in the meta-analysis).	2
Data collection process	10	Describe method of data extraction from reports (e.g., piloted forms, independently, in duplicate) and any processes for obtaining and confirming data from investigators.	2
Data items	11	List and define all variables for which data were sought (e.g., PICOS, funding sources) and any assumptions and simplifications made.	2
Risk of bias in individual studies	12	Describe methods used for assessing risk of bias of individual studies (including specification of whether this was done at the study or outcome level), and how this information is to be used in any data synthesis.	2-3

Section/topic	#	Checklist item	Reported on page #
Summary measures	13	State the principal summary measures (e.g., risk ratio, difference in means).	4
Synthesis of results	14	Describe the methods of handling data and combining results of studies, if done, including measures of consistency (e.g., I^2) for each meta-analysis.	4
Risk of bias across studies	15	Specify any assessment of risk of bias that may affect the cumulative evidence (e.g., publication bias, selective reporting within studies).	4
Additional analyses	16	Describe methods of additional analyses (e.g., sensitivity or subgroup analyses, meta-regression), if done, indicating which were pre-specified.	4
**RESULTS**			
Study selection	17	Give numbers of studies screened, assessed for eligibility, and included in the review, with reasons for exclusions at each stage, ideally with a flow diagram.	4<br>Fig. 1
Study characteristics	18	For each study, present characteristics for which data were extracted (e.g., study size, PICOS, follow-up period) and provide the citations.	4<br>Table 1
Risk of bias within studies	19	Present data on risk of bias of each study and, if available, any outcome level assessment (see item 12).	Table S1<br>Fig. S1
Results of individual studies	20	For all outcomes considered (benefits or harms), present, for each study: (a) simple summary data for each intervention group (b) effect estimates and confidence intervals, ideally with a forest plot.	Fig. 2-4<br>Fig. S2-6
Synthesis of results	21	Present results of each meta-analysis done, including confidence intervals and measures of consistency.	Fig. 2-4<br>Fig. S2-6
Risk of bias across studies	22	Present results of any assessment of risk of bias across studies (see Item 15).	Fig. S8
Additional analysis	23	Give results of additional analyses, if done (e.g., sensitivity or subgroup analyses, meta-regression [see Item 16]).	Fig. 2<br>Fig. S4, S7
**DISCUSSION**			
Summary of evidence	24	Summarize the main findings including the strength of evidence for each main outcome; consider their relevance to key groups (e.g., healthcare providers, users, and policy makers).	10
Limitations	25	Discuss limitations at study and outcome level (e.g., risk of bias), and at review-level (e.g., incomplete retrieval of identified research, reporting bias).	11
Conclusions	26	Provide a general interpretation of the results in the context of other evidence, and implications for future research.	10-12
**FUNDING**			

Section/topic	#	Checklist item	Reported on page #
Funding	27	Describe sources of funding for the systematic review and other support (e.g., supply of data); role of funders for the systematic review.	12

From: Moher D, Liberati A, Tetzlaff J, Altman DG, The PRISMA Group (2009). Preferred Reporting Items for Systematic Reviews and Meta-Analyses: The PRISMA Statement. PLoS Med 6(6): e1000097. doi:10.1371/journal.pmed1000097

For more information, visit: **www.prisma-statement.org**.

Appendix 2. PROSPERO registration

(CRD42021261414, please see next page.)

Citation

Xueyang Zhang, Lianhan Shang, Guohui Fan, Bin Cao. The efficacy and safety of Janus kinase (JAK) inhibitor treatment for COVID-19 patients: a living systemic review and meta-analysis. PROSPERO 2021 CRD42021261414 Available from:
https://www.crd.york.ac.uk/prospero/display_record.php?ID=CRD42021261414

Review question

(1) Does the JAK inhibitor treatment improve the clinical outcomes of COVID-19 patients?

(2) Does the JAK inhibitor treatment increase the incidence of adverse events in COVID-19 patients?

Searches

Literature searches in electronic databases was initiated on 6/11/2021: MEDLINE (via OVID), EMBASE, The Cochrane Central Register of Controlled Trials (CENTRAL), China national knowledge infrastructure (CNKI), Wanfang database, SinoMed, WHO COVID-19 database (Global literature on coronavirus disease), Cochrane COVID-19 study register. We also hand searched pre-print servers Medrxiv, Biorxiv, SSRN. The search strategy is built based on terms related to COVID-19, SARS-CoV-2, and Janus kinase inhibitors (including Ruxolitinib, Baricitinib, Tofacitinib, Upadacitinib, Fedratinib, Filgotinib, Peficitinib, Pacritinib, Solcitinib, Abrocitinib, and SHR0302).

There are no limitations on publication status or language.

As a living systemic review and meta-analysis, searches on databases will be conducted monthly. If important evidence which may potentially change the conclusion of the systematic review is published (e.g., RCT; non-randomized clinical trial or observational studies with large sample), this review will be updated in time.

Types of study to be included

Randomized controlled trials (RCT), non-randomized clinical trials, and observational studies with concurrent control.

Condition or domain being studied

Coronavirus Disease 2019 (COVID-19)

Participants/population

COVID-19 patients. There are no restrictions on age, races, occupation, economy or social status, religion, country, underlying conditions etc.

Intervention(s), exposure(s)

JAK inhibitor treatment: either Ruxolitinib, Baricitinib, Tofacitinib, Upadacitinib, Fedratinib, Filgotinib, Peficitinib, Pacritinib, Solcitinib, Abrocitinib, SHR0302, or other type of JAK inhibitor, or in combination of the above inhibitors.

Comparator(s)/control

(1) JAK inhibitor vs placebo. (2) JAK inhibitor vs standard of care.

Context

Exclusion criteria:

(1) Observational studies lacking concurrent control, case-series or case report studies, and all studies other than original research articles.

(2) In vitro and animal studies.

(3) Abstract only articles and articles with no full-text available.

(4) Studies not providing sufficient information to be included in the review.

Main outcome(s)

For question (1): mortality, incidence of ICU admission, incidence of invasive mechanical ventilation, time to recovery, duration of hospitalization.

For question (2): adverse events, serious adverse events, infection or secondary infection.

Measures of effect

Risk ratio (RR) or odds ratio (OR) with 95% confidence interval for dichotomous data.

Mean difference (MD) with 95% confidence interval for continuous data.

Additional outcome(s)

For question (1): time to virus clearance, inflammatory markers level, chest X-ray or computed tomography presentation, respiratory function.

Measures of effect

Risk ratio (RR) or odds ratio (OR) with 95% confidence interval for dichotomous data.

Mean difference (MD) with 95% confidence interval for continuous data.

Data extraction (selection and coding)

Two reviewers will independently perform study selection and data extraction work, and any disagreement will be resolved through discussion or consultation with a third reviewer.

Study selection from electronic databases output includes following steps: (1) duplicates removement; (2) title and abstract screening for potentially eligible studies; (3) full-text screening of studies from step (2).

Following information will be extracted from included studies: (1) authors; (2) study design; (3) publication time; (4) region; (5) inclusion criteria and exclusion criteria; (6) sample size; (7) intervention (type of JAK inhibitor, dose, route of administration, frequency, duration, other concurrent treatment); (8) treatment of control group; (9) key efficacy and safety outcomes.

Risk of bias (quality) assessment

Two reviewers will independently perform risk of bias assessment, and any disagreement will be resolved through discussion or consultation with a third reviewer.

(1) For RCT, Cochrane risk-of-bias 2.0 tool for randomized trials (RoB2) will be used.

(2) For non-randomized clinical trials, Risk of bias in non-randomized studies - of interventions (ROBINS-I) will be used.

(3) For observational studies, Newcastle Ottawa scale (NOS) will be used.

Strategy for data synthesis

Information extracted from the included studies will be narratively described. If adequate data are available for quantitative synthesis, a meta-analysis will be performed.

Risk ratio (RR) or odds ratio (OR) with 95% confidence interval will be adopted for dichotomous data, and Mean difference (MD) with 95% confidence interval for continuous data. If necessary, the original data will be converted by statistical methods to appropriate form for data synthesis. Based on the similarity of studies' methodology, either random-effects models or fixed-effect models will be used to pool the data. Heterogeneity among the studies will be assessed using the I^2 statistic. The sensitivity analysis will also be performed.

If quantitative analysis is not applicable due to the heterogeneity or any other reasons, a descriptive synthesis will be provided.

Analysis of subgroups or subsets
Subgroup analysis will be conducted if related data are available. Potential variables for sub-group analysis include study design, type of JAK inhibitors, baseline respiratory support, baseline inflammatory marker level (including d-dimer, c-reactive protein, ferritin, lactate dehydrogenase), comorbidities, time from symptoms onset to randomization, concurrent corticosteroid treatment, region, etc.

Contact details for further information
Bin Cao
caobin_ben@163.com

Organisational affiliation of the review
China-Japan Friendship Hospital

Review team members and their organisational affiliations
Ms Xueyang Zhang. Tsinghua University
Mr Lianhan Shang. Beijing University of Chinese Medicine
Mr Guohui Fan. China-Japan Friendship Hospital
Professor Bin Cao. China-Japan Friendship Hospital

Type and method of review
Intervention, Living systematic review, Meta-analysis, Systematic review

Anticipated or actual start date
11 June 2021

Anticipated completion date
31 July 2022

Funding sources/sponsors
Chinese Academy of Medical Sciences Innovation Fund for Medical Sciences (2018-I2M-1-003 and 2020-I2M-CoV19-005); National Natural Science Foundation of China (82041011)

Conflicts of interest

Language
English

Country
China

Stage of review
Review Completed not published

Subject index terms status
Subject indexing assigned by CRD

Subject index terms
COVID-19; Humans; Janus Kinase Inhibitors; Janus Kinases; Protein Kinase Inhibitors; Pyrimidines; SARS-CoV-2

Date of registration in PROSPERO
18 June 2021

Date of first submission
18 June 2021

Stage of review at time of this submission

Preliminary searches	Yes	Yes
Piloting of the study selection process	Yes	Yes
Formal screening of search results against eligibility criteria	Yes	Yes
Data extraction	Yes	Yes
Risk of bias (quality) assessment	Yes	Yes
Data analysis	Yes	Yes

The record owner confirms that the information they have supplied for this submission is accurate and complete and they understand that deliberate provision of inaccurate information or omission of data may be construed as scientific misconduct.
The record owner confirms that they will update the status of the review when it is completed and will add publication details in due course.

Versions

18 June 2021
27 June 2021

Appendix 3. Search strategies

Medline via Ovid

1 COVID-19.sh.

2 (COVID-19 or COVID 19 or COVID19 or COVID-19 Virus Disease or COVID 19 Virus Disease or COVID-19 Virus Diseases or COVID-19 Virus Infection or COVID 19 Virus Infection or COVID-19 Virus Infections or 2019-nCoV Infection or 2019 nCoV Infection or 2019-nCoV Infections or 2019-nCoV Disease or 2019 nCoV Disease or 2019-nCoV Diseases or Coronavirus Disease-19 or Coronavirus Disease 19 or Coronavirus Disease 2019 or 2019 Novel Coronavirus Disease or 2019 Novel Coronavirus Infection or SARS Coronavirus 2 Infection or SARS-CoV-2 Infection or SARS CoV 2 Infection or SARS-CoV-2 Infections or COVID-19 Pandemic or COVID 19 Pandemic or COVID-19 Pandemics).ab,kf,ot,ti,fx.

3 SARS-COV-2.sh.

4 (SARS-CoV-2 or Severe Acute Respiratory Syndrome Coronavirus 2 or Coronavirus Disease 2019 Virus or 2019 Novel Coronavirus or 2019 Novel Coronaviruses or SARS-CoV-2 Virus or SARS CoV 2 Virus or SARS-CoV-2 Viruses or 2019-nCoV or COVID-19 Virus or COVID 19 Virus or COVID-19 Viruses or SARS Coronavirus 2).ab,kf,ot,ti,fx.

5 Janus kinase inhibitors.sh.

6 (JAK inhibitor* or Janus kinase inhibitor* or Ruxolitinib or Jakafi or Jakavi or INCB018424 or INC424 or Baricitinib or Olumiant or INCB28050 or LY3009104 or Tofacitinib or Xeljanz or Jakvinus or CP-690550 or Upadacitinib or Ronviq or ABT494 or ABT-494 or Fedratinib or Inrebic or GLPG0634 or Filgotinib or GLPG0634 or Peficitinib or ASP015K or Pacritinib or Solcitinib or GLPG0778 or GSK2586184 or SHR0302 or Abrocitinib or PF-04965842).ab,kf,ot,ti,fx.

7 1 or 2 or 3 or 4

8 5 or 6

9 7 and 8

Embase

#1 'coronavirus disease 2019'/exp

#2 'covid-19':ta,ab,kw OR 'covid 19':ta,ab,kw OR 'covid19':ta,ab,kw OR 'covid-19 virus disease':ta,ab,kw OR 'covid 19 virus disease':ta,ab,kw OR 'covid-19 virus diseases':ta,ab,kw OR 'covid-19 virus infection':ta,ab,kw OR 'covid 19 virus infection':ta,ab,kw OR 'covid-19 virus infections':ta,ab,kw OR '2019-ncov infection':ta,ab,kw OR '2019 ncov infection':ta,ab,kw OR '2019-ncov infections':ta,ab,kw OR '2019-ncov disease':ta,ab,kw OR '2019 ncov disease':ta,ab,kw OR '2019-ncov diseases':ta,ab,kw OR 'coronavirus disease-19':ta,ab,kw OR 'coronavirus disease 19':ta,ab,kw OR 'coronavirus disease 2019':ta,ab,kw OR '2019 novel coronavirus disease':ta,ab,kw OR '2019 novel coronavirus infection':ta,ab,kw OR 'sars coronavirus 2 infection':ta,ab,kw OR 'sars-cov-2 infection':ta,ab,kw OR 'sars cov 2 infection':ta,ab,kw OR 'sars-cov-2 infections':ta,ab,kw OR 'covid-19 pandemic':ta,ab,kw OR 'covid 19 pandemic':ta,ab,kw OR 'covid-19 pandemics':ta,ab,kw

#3 'severe acute respiratory syndrome coronavirus 2'/exp

#4 'sars-cov-2':ta,ab,kw OR 'severe acute respiratory syndrome coronavirus 2':ta,ab,kw OR 'coronavirus disease 2019 virus':ta,ab,kw OR '2019 novel coronavirus':ta,ab,kw OR '2019 novel coronaviruses':ta,ab,kw OR 'sars-cov-2 virus':ta,ab,kw OR 'sars cov 2 virus':ta,ab,kw OR 'sars-cov-2 viruses':ta,ab,kw OR '2019-ncov':ta,ab,kw OR 'covid-19 virus':ta,ab,kw OR 'covid 19 virus':ta,ab,kw OR 'covid-19 viruses':ta,ab,kw OR 'sars coronavirus 2':ta,ab,kw

#5 'janus kinase inhibitor'/exp

#6 'jak inhibitor*':ta,ab,kw OR 'janus kinase inhibitor*':ta,ab,kw OR ruxolitinib:ta,ab,kw OR jakafi:ta,ab,kw OR jakavi:ta,ab,kw OR incb018424:ta,ab,kw OR inc424:ta,ab,kw OR baricitinib:ta,ab,kw OR olumiant:ta,ab,kw OR incb28050:ta,ab,kw OR ly3009104:ta,ab,kw OR tofacitinib:ta,ab,kw OR xeljanz:ta,ab,kw OR jakvinus:ta,ab,kw OR 'cp-690550':ta,ab,kw OR upadacitinib:ta,ab,kw OR ronviq:ta,ab,kw OR abt494:ta,ab,kw OR 'abt-494':ta,ab,kw OR fedratinib:ta,ab,kw OR inrebic:ta,ab,kw OR filgotinib:ta,ab,kw OR glpg0634:ta,ab,kw OR peficitinib:ta,ab,kw OR

asp015k:ta,ab,kw OR pacritinib:ta,ab,kw OR solcitinib:ta,ab,kw OR glpg0778:ta,ab,kw OR gsk2586184:ta,ab,kw OR shr0302:ta,ab,kw OR abrocitinib:ta,ab,kw OR 'pf-04965842':ta,ab,kw
#7 #1 OR #2 OR #3 OR #4
#8 #5 OR #6
#9 #7 AND #8

The Cochrane Central Register of Controlled Trials (CENTRAL)
#1 MeSH descriptor: [COVID-19] explode all trees
#2 COVID-19 OR COVID 19 OR COVID19 OR COVID-19 Virus Disease OR COVID 19 Virus Disease OR COVID-19 Virus Diseases OR COVID-19 Virus Infection OR COVID 19 Virus Infection OR COVID-19 Virus Infections OR 2019 nCoV Infection OR 2019 nCoV Infections OR 2019 nCoV Disease OR 2019 nCoV Disease OR 2019 nCoV Diseases OR Coronavirus Disease-19 OR Coronavirus Disease 19 OR Coronavirus Disease 2019 OR 2019 Novel Coronavirus Disease OR 2019 Novel Coronavirus Infection OR SARS Coronavirus 2 Infection OR SARS-CoV-2 Infection OR SARS CoV 2 Infection OR SARS-CoV-2 Infections OR COVID-19 Pandemic OR COVID 19 Pandemic OR COVID-19 Pandemics
#3 MeSH descriptor: [SARS-CoV-2] explode all trees
#4 SARS-CoV-2 OR Severe Acute Respiratory Syndrome Coronavirus 2 OR Coronavirus Disease 2019 Virus OR 2019 Novel Coronavirus OR 2019 Novel Coronaviruses OR SARS-CoV-2 Virus OR SARS CoV 2 Virus OR SARS-CoV-2 Viruses OR 2019 nCoV OR COVID-19 Virus OR COVID 19 Virus OR COVID-19 Viruses OR SARS Coronavirus 2
#5 MeSH descriptor: [Janus Kinase Inhibitors] explode all trees
#6 JAK inhibitor* or Janus kinase inhibitor* or Ruxolitinib or Jakafi or Jakavi or INCB018424 or INC424 or Baricitinib or Olumiant or INCB28050 or LY3009104 or Tofacitinib or Xeljanz or Jakvinus or CP-690550 or Upadacitinib or Ronviq or ABT494 or ABT-494 or Fedratinib or Inrebic or GLPG0634 or Filgotinib or GLPG0634 or Peficitinib or ASP015K or Pacritinib or Solcitinib or GLPG0778 or GSK2586184 or SHR0302 or Abrocitinib or PF-04965842
#7 #1 OR #2 OR #3 OR #4
#8 #5 OR #6
#9 #7 AND #8 in Trials

China national knowledge infrastructure (CNKI)
（全文：冠状病毒肺炎 + 2019 冠状病毒病 + 新冠肺炎 + 新冠病毒 + 新型冠状病毒 + 严重急性呼吸综合征冠状病毒 2 + 'SARS-COV-2' + 'COVID-19' + '2019-ncov' + 'COVID-2019'） AND （全文：JAK 抑制剂 + Janus 激酶抑制剂 + 'JAK inhibitor*' + 'Janus kinase inhibitor*'）

Wanfang Database
全部:(冠状病毒肺炎 OR 2019 冠状病毒病 OR 新冠肺炎 OR 新冠病毒 OR 新型冠状病毒 OR 严重急性呼吸综合征冠状病毒 2 OR SARS-COV-2 OR COVID-19 OR 2019-ncov OR COVID-2019) and 全部:(JAK 抑制剂 OR Janus 激酶抑制剂 OR 'JAK inhibitor' OR 'Janus kinase inhibitor' OR 'JAK inhibitors' OR 'Janus kinase inhibitors')

Sinomed
("冠状病毒肺炎"[常用字段:智能] OR "2019 冠状病毒病"[常用字段:智能] OR "新冠肺炎"[常用字段:智能] OR "新冠病毒"[常用字段:智能] OR "新型冠状病毒"[常用字段:智能] OR "严重急性呼吸综合征冠状病毒 2"[常用字段:智能] OR "SARS-COV-2"[常用字段:智能] OR "COVID-19"[常用字段:智能] OR "2019-ncov"[常用字段:智能] OR "COVID-2019"[常用字段:智能]) AND("JAK 抑制剂"[常用字段:智能] OR "Janus 激酶抑制剂"[常用字段:智能] OR "JAK inhibitor"[常用字段:智能] OR "Janus kinase inhibitor"[常用字段:智能] OR "JAK inhibitors"[常用字段:智能] OR "Janus kinase inhibitors"[常用字段:智能])

WHO COVID database (Global literature on coronavirus disease database)

"JAK inhibitor" OR "JAK inhibitors" OR "Janus kinase inhibitor" OR "Janus kinase inhibitors" OR Ruxolitinib OR Baricitinib OR Tofacitinib OR Upadacitinib OR Fedratinib OR Filgotinib OR Peficitinib OR Pacritinib OR Solcitinib OR Abrocitinib

Cochrane COVID-19 study register

"JAK inhibitor" OR "JAK inhibitors" OR "Janus kinase inhibitor" OR "Janus kinase inhibitors" OR Ruxolitinib OR Baricitinib OR Tofacitinib OR Upadacitinib OR Fedratinib OR Filgotinib OR Peficitinib OR Pacritinib OR Solcitinib OR Abrocitinib

Hand-searched preprint servers: MedRxiv, BioRxiv, SSRN
